# Sestrins are evolutionarily conserved mediators of exercise benefits

**DOI:** 10.1038/s41467-019-13442-5

**Published:** 2020-01-13

**Authors:** Myungjin Kim, Alyson Sujkowski, Sim Namkoong, Bondong Gu, Tyler Cobb, Boyoung Kim, Allison H. Kowalsky, Chun-Seok Cho, Ian Semple, Seung-Hyun Ro, Carol Davis, Susan V. Brooks, Michael Karin, Robert J. Wessells, Jun Hee Lee

**Affiliations:** 1 Department of Molecular & Integrative Physiology, University of Michigan, Ann Arbor, MI 48109 USA; 2 Department of Physiology, Wayne State University School of Medicine, Detroit, MI 48201 USA; 3 Department of Pharmacology, University of California San Diego, La Jolla, CA 92093 USA; 4 Present Address: Department of Biochemistry, College of Natural Sciences, Kangwon National University, Chuncheon, Gangwon 24341 Republic of Korea; 5 Present Address: Department of Biochemistry, University of Nebraska, Lincoln, NE 68588 USA

**Keywords:** Metabolism, Energy metabolism, Molecular medicine

## Abstract

Exercise is among the most effective interventions for age-associated mobility decline and metabolic dysregulation. Although long-term endurance exercise promotes insulin sensitivity and expands respiratory capacity, genetic components and pathways mediating the metabolic benefits of exercise have remained elusive. Here, we show that Sestrins, a family of evolutionarily conserved exercise-inducible proteins, are critical mediators of exercise benefits. In both fly and mouse models, genetic ablation of Sestrins prevents organisms from acquiring metabolic benefits of exercise and improving their endurance through training. Conversely, Sestrin upregulation mimics both molecular and physiological effects of exercise, suggesting that it could be a major effector of exercise metabolism. Among the various targets modulated by Sestrin in response to exercise, AKT and PGC1α are critical for the Sestrin effects in extending endurance. These results indicate that Sestrin is a key integrating factor that drives the benefits of chronic exercise to metabolism and physical endurance.

## Abstract (web summary)

Exercise improves metabolic health and physical condition, particularly important for health in aged individuals. Here, the authors identify that Sestrins, proteins induced by exercise, are key mediators of the metabolic adaptation to exercise and increase endurance through the AKT and PGC1a axes.

## Introduction

As the percentage of elderly members in the population continues to increase, concerns about increased health care responsibilities have been amplified as well. Interventions that can preserve proper body function at advanced ages are thus of substantial importance. In repeated surveys, elderly members of the community have mentioned preservation of mobility as their biggest concern associated with aging^1^. Mobility is important both for direct health reasons (e.g. preventing falls, retaining access to relatives and health care providers) and for psychological reasons, as it is highly correlated with retained personal satisfaction and morale^2,3^. Identification of cost-effective therapies that can preserve independence and healthy mobile capacity in the elderly would therefore have a cumulatively positive effect, influencing many other aspects of mental and physical health in a wide population demographic.

One promising therapeutic intervention to impede age-related functional decline is endurance exercise. Endurance training induces remodeling in muscle tissue that alters the metabolic health of the entire organism^4–6^. Evidence from humans and model organisms strongly suggests that endurance exercise has substantially protective effects on various indices of healthspan^7^. In vertebrates, endurance training leads to increased mitochondrial biogenesis/efficiency^8^, decreased triglyceride storage^9^, improved insulin sensitivity^10^, and protection of both muscle and neural functions^11^. These changes are often thought to be at least partially mediated by exercise-induced upregulation of AMP-activated protein kinase (AMPK) and the insulin-AKT pathway^12^. These remodeling events lead to increased physiological capacity in both cardiac and skeletal muscle. However, substantial obstacles exist against the widespread application of endurance therapy in the human population. Many humans are unable to reach the level of training necessary to generate these remodeling steps for several reasons: (1) age, (2) injury, (3) illness, or (4) social commitments that require long sedentary periods. Therefore, generation of therapeutic mimetics to induce the benefits of exercise could provide broad ranging benefits to the medical community.

Sestrins are small stress-inducible proteins that are found throughout the animal kingdom^13^. Mammals express three Sestrins (Sesn1-3), while *Drosophila* and *C. elegans* express one Sestrin orthologue (dSesn and cSesn, respectively)^14^. dSesn and mammalian Sesn1 are predominantly expressed in skeletal and cardiac muscles^14–16^. Importantly, Sestrin expression is further increased by exercise training in both humans and mice^17–19^. Once induced, Sestrins coordinate metabolic homeostasis by regulating multiple signaling pathways^20^. Through its intrinsic oxidoreductase activity and by regulating autophagy, Sestrin can function as an antioxidant to reduce oxidative damage in cells^20^. In addition, through activation of AMPK^21^ and modulation of GAP activity towards Rags (GATOR) protein complexes^22–24^, Sestrins inhibit target of rapamycin complex 1 (TORC1)/S6 kinase (S6K) signaling. Importantly, while Sestrins downregulate TORC1/S6K signaling, they strongly activate TORC2/AKT signaling^25^, independently of the TORC1 regulation^26^. Interestingly, exercise-inducible Sestrins activate both AMPK and AKT^20^, effectors that are also upregulated by endurance exercise training^12^. Indeed, upregulation of AMPK signaling and insulin-TORC2/AKT mediates the protective activities of Sestrins against insulin resistance and diabetes^25,26^. However, none of the former studies examined the actual genetic and physiological roles of Sestrins in the response to exercise.

Here, using Sestrin-deficient fly and mouse models, we show that Sestrins play a critical role in mediating chronic exercise adaptations and exercise benefits. Sestrins, acting through multiple effector molecules such as AKT and PGC1α, are both necessary and sufficient to produce exercise effects on improving muscle metabolism, functionality, and endurance.

## Results

### Sestrin is required for increased performance after exercise

**Fig. 1:**
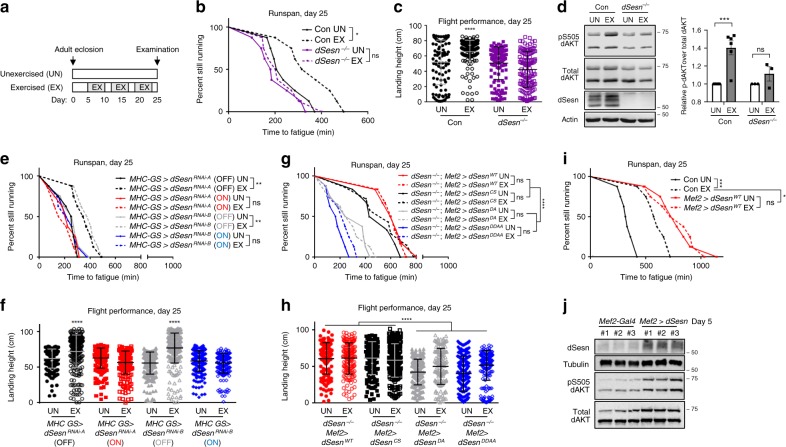
Sestrin is critical for exercise response in *Drosophila*. **a** Schematic of exercise training in *Drosophila*. Gray boxes indicate days with control (UN) or exercise (EX) treatments. **b**–**d** Wild-type (Con; *w*^*1118*^) and *dSesn*-null (*dSesn*^*−/−*^) flies were analyzed. **e**, **f** Two independent RNAi constructs targeting dSesn were conditionally expressed in muscle tissue using myosin heavy chain-gene switch (*MHC-GS*) driver, controlled by mifepristone feeding (ON/OFF). **g**–**j** Wild-type, C86S, D423A, D423A/D424A dSesn transgenes were expressed in muscle of *dSesn*^*−/−*^ flies (**g**, **h**) or WT flies (**i**). C86, D423 and D424 in dSesn correspond to C125, D406 and D407 in Sesn2. Flies were analyzed by runspan (**b**, **e**, **g**, **i**) or flight performance (**c**, **f**, **h**) assays. In flight performance graphs, closed and open shapes indicate individual data from control (UN) and exercised (EX) experiments, respectively. Endogenous dSesn was overexpressed through EP line version of *UAS-dSesn* (**j**). Leg and thoracic muscle were analyzed by immunoblotting (**d, j**) and band densitometry (**d**, right; values relative to corresponding UN levels). Biologically independent animal groups: *n* = 8 (**b**, **e**, **i**; groups of 20 flies), *n* = 6, 6, 3, 3 (**d**, left to right; groups of 20 flies), *n* = 6, 6, 6, 7, 8, 8, 8, 8 (**g**, upper to lower; groups of 20 flies). Biologically independent animals: *n* = 100, 193, 141, 145 (**c**, left to right), *n* = 196, 264, 250, 233, 257, 288, 218, 234 (**f**, left to right), *n* = 149, 159, 144, 187, 223, 148, 233, 237 (**h**, left to right). Error bars, s.d. (flight performance) or s.e.m. (immunoblot quantification). *P < 0.05, **P < 0.01, ***P < 0.001, ****P < 0.0001 or ns, non-significant from a log-rank test (runspan) or two-tailed student’s *t* test (all other assays). Molecular weight markers are indicated in kDa.

We established a protocol for endurance training and measuring physical endurance in *Drosophila*, which represents the first chronic exercise model in an invertebrate system^27^. Three weeks of exercise training (Fig. 1a) substantially extended running endurance (runspan, length of time that vials of flies are able to run in response to stimulation; Fig. 1b) and flight performance (landing height after ejection into a space; Fig. 1c) of wild-type (WT) flies, consistent with earlier studies^27^. These changes were accompanied by a strong induction of dSesn expression and increased TORC2-dependent AKT phosphorylation (Fig. 1d and Supplementary Fig. 1a–c). Although exercise increased AMPK phosphorylation, it did not significantly affect TORC1-dependent S6K phosphorylation in muscle (Supplementary Fig. 1a, d, e). By contrast, exercise did not increase running endurance, flight performance and AKT phosphorylation in *dSesn*-null flies (Fig. 1b–d). These results indicate that Sestrin is necessary for improving endurance and flight performance after exercise.

### Sestrin in muscle is sufficient to modify exercise outcome

**Fig. 2:**
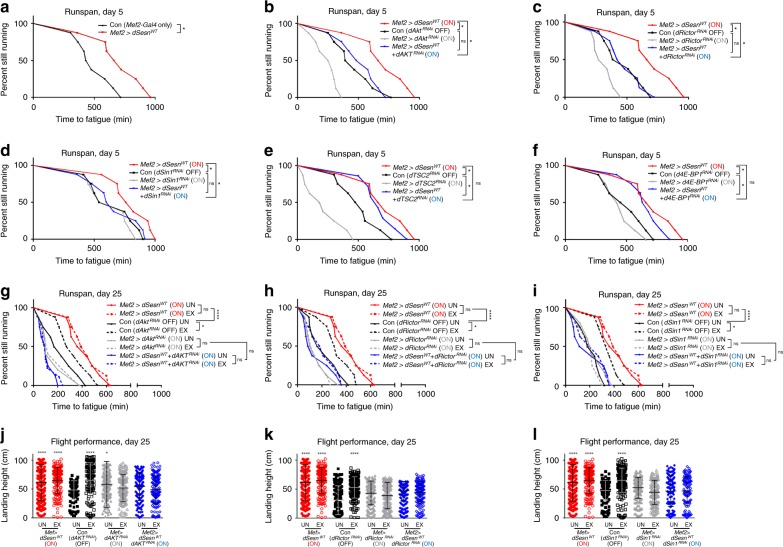
Exercise and Sestrin improve endurance through the TORC2-AKT axis. **a**–**f** Runspan assays were conducted using indicated 5-day-old fly strains. **g**–**l** Flies were subjected to control (UN) or exercise (EX) regimen. Runspan and flight performance assays were conducted at day 25. In flight performance graphs, closed and open shapes indicate individual data from control (UN) and exercised (EX) experiments, respectively. ON indicates that transgenes are activated by the presence of *Mef2-Gal4* driver. OFF indicates that UAS transgenes are inactive due to the absence of Gal4. *Mef2* *>* *dSesn*^*WT*^ (ON) data from the same cohort are shown in each graph as a reference. Biologically independent animal groups: *n* = 8 (**a**, **b**, **c**, **d**, **f**, **h**; groups of 20 flies), *n* = 8, 8, 8, 7 (**e**, upper to lower; groups of 20 flies), *n* = 8, 8, 8, 8, 5, 5, 8, 8 (**g**, upper to lower; groups of 20 flies), *n* = 8, 8, 8, 8, 5, 4, 8, 8 (**i**, upper to lower; groups of 20 flies). Biologically independent animals: *n* = 227, 172, 139, 113, 165, 152, 155, 156 (**j**, left to right), 227, 172, 128, 171, 178, 164, 250, 164 (**k**, left to right), 227, 172, 166, 212, 197, 164, 127, 115 (**l**, left to right). Error bars, s.d. *P < 0.05, ****P < 0.0001 or ns, non-significant from a log-rank test (runspan, between indicated groups) or two-tailed student’s *t* test (flight performance, compared to Con-UN group).

Because Sestrin expression is high and exercise-inducible in muscle, we tested whether Sestrin functions in muscle to mediate exercise. Muscle-specific dSesn silencing through two independent *dSesn*^*RNAi*^ constructs (Supplementary Fig. 1f) nullified the exercise benefits to the same degree as complete dSesn ablation (Fig. 1e, f). Conversely, muscle-specific transgenic dSesn overexpression in *dSesn*-null flies (Supplementary Fig. 1g) was sufficient to increase both runspan and flight performance, even in the absence of exercise (Fig. 1g, h). These results indicate that (1) Sestrin functions in muscle to mediate exercise-induced increase of mobility and (2) Sestrin upregulation in muscle was sufficient to induce the benefits of exercise. Indeed, muscle-specific Sestrin overexpression in WT flies produced strong extension of running endurance (Figs. 1i and 2a) associated with AKT activation (Fig. 1j). Importantly, the effect of dSesn overexpression was quantitatively greater than the effect of exercise, and exercise training did not improve running endurance in Sestrin-overexpressing flies (Fig. 1g–i). These results indicate that muscle-specific Sestrin induction is both necessary and sufficient for mediating the endurance benefit of chronic exercise.

### Sestrin improves endurance through TOR modulation

We next examined how Sestrin expression improves endurance and flight performance mechanistically. The C86S mutation in Sestrin, which disrupts its oxidoreductase function^20^, slightly reduced Sestrin’s capability in extending endurance (Fig. 1g, h). In contrast, the D423A and D424A substitutions, which abolish Sestrin’s effects on TORC1 inhibition and TORC2/AKT activation^20^ (Supplementary Fig. 1h–o), almost completely blocked the Sestrin effect (Fig. 1g, h), indicating that regulation of the TOR complexes is important for Sestrin’s beneficial effects.

The transgenic expression levels of Sestrin proteins with C86S or D423A/D424A mutation were comparable to that of wild-type Sestrin in immunoblotting (Supplementary Fig. 1g) and immunostaining (Supplementary Fig. 1l–o) experiments. When expressed in developing wing imaginal discs, wild-type and C86S Sestrins reduced TORC1-dependent organ growth (Supplementary Fig. 1h–m) while activating TORC2-dependent AKT phosphorylation (Supplementary Fig. 1j–m). In contrast, D423A/D424A mutant Sestrins did not affect organ growth or AKT phosphorylation (Supplementary Fig. 1h–o). These results confirm that D423 and D424 residues are indeed critical for Sestrin-dependent modulation of TOR complexes.

### TORC2-AKT axis is required for Sestrin to extend endurance

Like exercise, Sestrin strongly upregulated TORC2-mediated AKT phosphorylation (Fig. 1j)^20,26,28^. Inhibition of TORC2 components, such as Rictor and Sin1 or AKT, completely blocked Sestrin’s effect in extending endurance (Fig. 2b–d). In contrast, inhibition of TSC2, which blocks Sestrin-mediated TORC1 inhibition^14^, or 4E-BP knockdown, which uncouples TORC1 signaling from translational regulation^29^, did not affect the ability of Sestrin to extend endurance (Fig. 2e, f). TORC2/AKT inhibition not only inhibited the Sestrin effect but also blocked the exercise effect (Fig. 2g–l). Therefore, the TORC2-AKT axis appears to be a critical effector of both exercise and Sestrin, while the traditional Sestrin target TORC1 is less important in this context.

### Exercise requires Sesn1 to improve insulin sensitivity

**Fig. 3:**
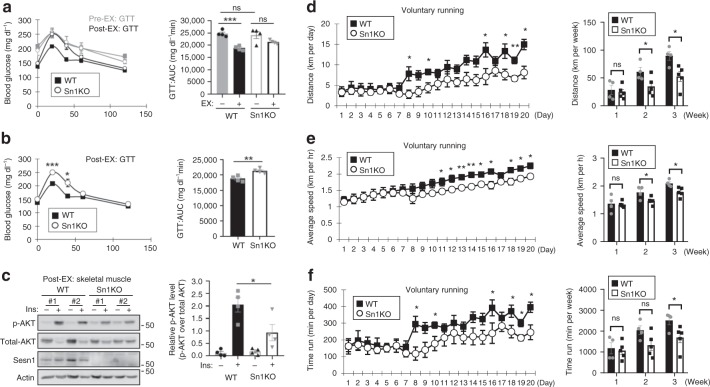
Sesn1 is required for exercise effects on glucose tolerance and running profile. **a**–**c** 2-month-old WT and *Sesn1*^*−/−*^ (Sn1KO) mice (C57BL/6 background) were analyzed by glucose tolerance test (GTT) with area-under-curve (AUC) analysis (**a**, Pre-EX). When the mice reached 6 months of age, they were subjected to voluntary wheel running for 2 months, and analyzed by GTT (**a**, **b**; Post-EX) and acute insulin response assay (**c**). Post-EX data were plotted with (**a**) or without (**b**) Pre-EX data for cleaner comparisons between groups. Insulin-induced AKT activation of skeletal muscle was analyzed by immunoblotting and band densitometry. **d**–**f** 6-month-old WT and *Sesn1*^*−/−*^ mice were subjected to voluntary wheel running. Daily (left panels) and weekly (right panels) running distance (**d**), speed (**e**) and time (**f**) were recorded for the first three weeks of voluntary wheel running. Biologically independent animals: *n* = 4 (**a**–**c**), *n* = 4, 5 (**d**–**f**; WT and *Sesn1*^*−/−*^ mice, respectively). Error bars, s.e.m. *P < 0.05, **P < 0.01, ***P < 0.001 or ns, non-significant from a two-tailed student’s *t* test. Molecular weight markers are indicated in kDa.

AKT signaling in mammals is important for insulin sensitivity and glucose metabolism. In mice, Sesn2 and Sesn3, which regulate insulin signaling in the liver, were shown to be important for glucose homeostasis^25,26^. But among the three mammalian Sestrins, Sesn1 is predominantly expressed in muscle^16^. Unlike *Sesn2*^*−/−*^ or *Sesn3*^*−/−*^ mice^25,26^, *Sesn1*^*−/−*^ mice did not show any detectable changes in glucose homeostasis in either lean or obese sedentary conditions (Fig. 3a and Supplementary Fig. 2a). In WT mice, voluntary wheel running improved glucose tolerance (Fig. 3a); however, this effect was attenuated in *Sesn1*^*−/−*^ mice (Fig. 3a) and exercised *Sesn1*^*−/−*^ mice showed higher glucose intolerance compared to the exercised WT counterparts (Fig. 3b). Insulin-induced AKT activation in trained skeletal muscle was also reduced in *Sesn1*^*−/−*^ mice (Fig. 3c). These results suggest that Sesn1 is involved in the muscle metabolic response to exercise.

### Exercise requires Sesn1 to improve running

Based on our observations from the fly model, we wanted to know if Sesn1 is also necessary for increasing running capacity. In WT mice, running distance, speed, and time were gradually increased in accordance with the duration of voluntary training (Fig. 3d–f). Although *Sesn1*^*−/−*^ mice initially ran at the same distance and speed, they did not improve their running profiles as efficiently as WT mice (Fig. 3d–f). The same effects of exercise and Sesn1 loss were observed in an independent and older cohort of mice (Supplementary Fig. 2b–d). Therefore, the role of Sestrin in mediating exercise benefits of improving physical movement and upregulating insulin-AKT signaling is conserved between mice and flies.

### Exercise requires Sesn1 to improve respiratory efficiency

Exercise training induces metabolic changes that allow for improved respiratory capacity^12^. To evaluate the effect Sestrin may have on this, mice were subjected to incremental exercise (forced running of increasing intensity) with respiratory assessment (Supplementary Fig. 3a). In untrained WT mice, there was a gradual increase in respiratory exchange ratio (RER; VCO_2_∙VO_2_^−1^) as the exercise load became more intense, while overall oxygen consumption rate (VO_2_) remained relatively constant (Supplementary Fig. 3b, c). This was associated with a change in energy source from fatty acids to glucose (Supplementary Fig. 3d)^12^. In contrast, wheel-trained WT mice maintained low RER and high fat oxidation during heavy exercise (Supplementary Fig. 3e–h). Notably, wheel-trained *Sesn1*^−/−^ mice did not show such metabolic improvements and exhibited a metabolic profile similar to untrained mice (Supplementary Fig. 3e–h). Compared to wheel-trained WT mice, RER of wheel-trained *Sesn1*^−/−^ mice was more strongly increased upon heavy exercise (Supplementary Fig. 3i), while fat oxidation was reduced (Supplementary Fig. 3j). Nevertheless, forced running endurance was not strongly reduced in *Sesn1*^*−/−*^ mice in terms of running time and distance (Supplementary Fig. 3k, l). In addition, exercise metabolism profiles were similar between untrained mice of WT and *Sesn1*^*−/−*^ (Supplementary Fig. 4a–f).

### *Sesn*-TKO impairs exercise metabolism and endurance

**Fig. 4:**
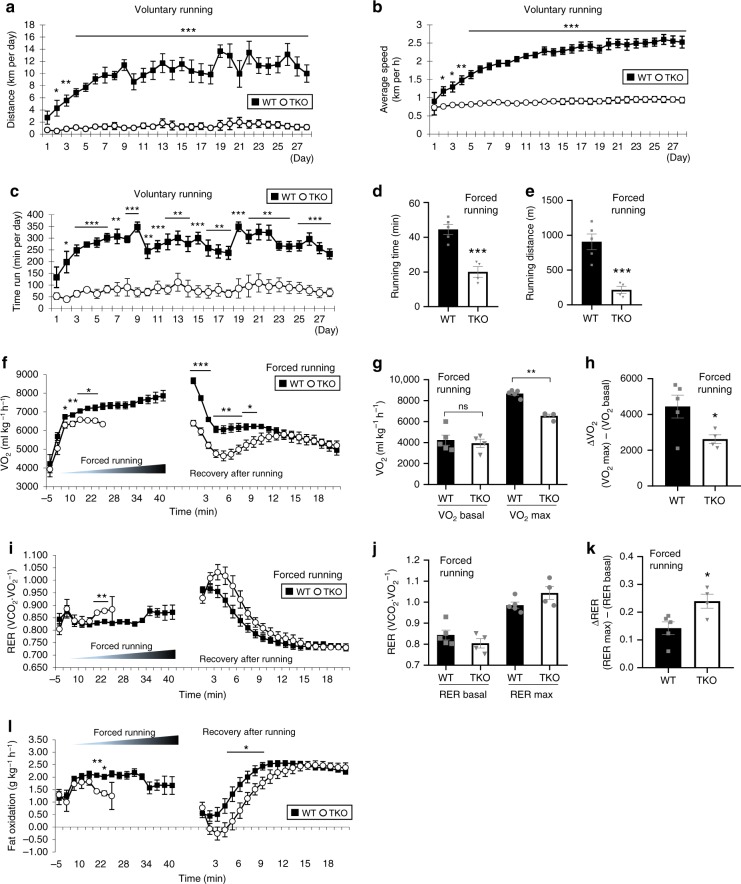
Sesn1-3 triple knockout mice display drastic impairment in exercise response. 5-month-old WT and *Sesn1*^*−/−*^/*Sesn2*^*−/−*^/*Sesn3*^*−/−*^ (TKO) mice (C57BL/6 background) were subjected to voluntary wheel running for 1 month. Running distance (**a**), speed (**b**) and time (**c**) were recorded. Mice were then subjected to incremental exercise (forced running of increasing intensity until exhaustion) with indirect calorimetry (**d**–**l**). VO_2_, oxygen consumption rate. VO_2_ basal, baseline VO_2_. VO_2_ max, maximum VO_2_. ΔVO_2_, VO_2_ max − VO_2_ basal. VCO_2_, CO_2_ production rate. RER, respiratory exchange ratio. RER basal, baseline RER. RER max, maximum RER. ΔRER, RER max – RER basal. Biologically independent animals: *n* = 5, 4 (**a**–**l**; WT and TKO mice, respectively). Error bars, s.e.m. *P < 0.05, **P < 0.01, ***P < 0.001 or ns, non-significant from a two-tailed student’s *t* test.

The modest decrease in exercise capacity of *Sesn1*^*−/−*^ mice could be due to the presence of Sesn2 and Sesn3, which are also expressed in muscle and induced upon exercise^16–19^. To eliminate all Sestrins, we generated *Sesn1/2/3* triple knockout (TKO) mice. When placed on training wheels, TKO mice did not show any improvements in running distance and speed (Fig. 4a–c). Although TKO mice showed almost normal metabolic profiles with unaltered body weight (Supplementary Fig. 5a), body composition (Supplementary Fig. 5b, c) and circadian rhythmicity in energy metabolism (Supplementary Fig. 5d, e), they exhibited a slight reduction in food consumption and physical activities (Supplementary Fig. 5f, g). When subjected to forced treadmill running, TKO mice performed very poorly with dramatically reduced running time and distance (Fig. 4d, e).

This endurance phenotype was associated with strong defects in respiratory metabolism. Rates of oxygen consumption (VO_2_) remained low throughout forced running and recovery (Fig. 4f), and maximal aerobic capacity (VO_2_ max) was substantially reduced in TKO mice while basal oxygen consumption rate was unaltered (Fig. 4g). Therefore, exercise-induced VO_2_ (ΔVO_2_) was almost cut in half in TKO mice (Fig. 4h). As observed in *Sesn1*^*−/−*^ mice, TKO mice also exhibited increased respiratory inefficiency (high RER; Fig. 4i–k) and decreased fat oxidation (Fig. 4l) during running and recovery. Notably, RER, a marker for metabolic exhaustion^30^, was strongly elevated right around exhaustion in both WT and TKO mice (Fig. 4i) and remained high during early recovery period (Fig. 4i), indicating that TKO mice did not simply choose to stop running but were metabolically exhausted. Notably, at time points when the Sestrin TKO mice were exhausting (around 20–25 min after running), the RER levels of Sestrin TKO mice were significantly higher than WT mice (Fig. 4i). Furthermore, RER induction during exercise (ΔRER) was substantially stronger in TKO mice compared to WT mice (Fig. 4j, k). These results indicate that defective running capacity in TKO mice is not due to a behavioral change but a metabolic alteration associated with respiratory defects.

Notably, TKO defects in forced running (Fig. 4d, e) and exercise metabolism (Fig. 4f–l) were very strong and not exhibited by any wild-type mouse cohorts. These cohorts include mice subjected to voluntary training (Fig. 4d–l), unexercised wild-type mice (Supplementary Fig. 3) and unexercised and aged wild-type mice (Supplementary Fig. 4). All these wild-type mice exhibited robust running performance and stable exercise metabolism at the low-speed running condition (12–19 m min^−1^), while TKO mice exhibited completely penetrant metabolic and physical exhaustion at the same condition. Therefore, the defects of TKO mice observed during forced running (Fig. 4d–l) are not simply because they did not get benefits from voluntary wheel training (Fig. 4a–c).

### *Sesn*-TKO reduces mitochondrial biogenesis in muscle

**Fig. 5:**
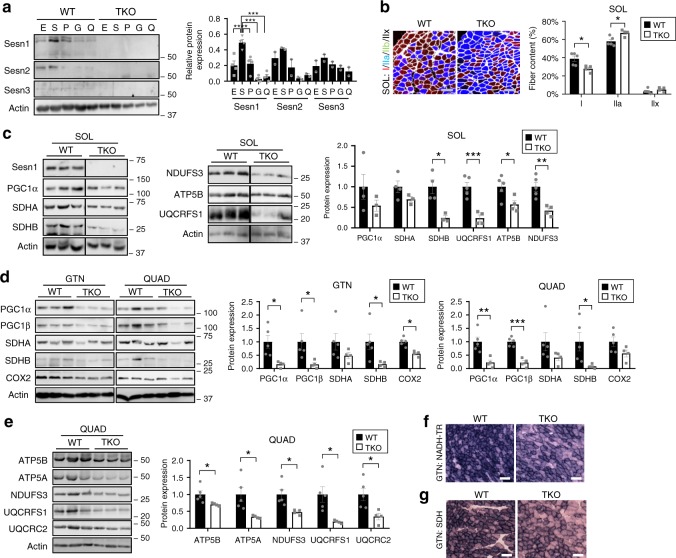
Sestrin regulates mitochondrial biogenesis in skeletal muscle. **a** Sestrin expression in different muscle tissue was analyzed by immunoblotting and band densitometry. E, extensor digitorum longus; S, soleus (SOL); P, plantaris; G, gastrocnemius (GTN); Q, quadriceps (QUAD). **b** Soleus muscle was analyzed by immunostaining visualizing extracellular matrix (white) and myofibers of type I (red), IIA (blue), IIB (green; SOL did not contain IIB fibers in our analysis) and IIX (black). Fiber contents of the whole cross-section were quantified (right). **c**–**e** Indicated muscle tissues were analyzed by immunoblotting and band densitometry. **f**, **g** Mitochondrial complex I (NADH-TR) and II (SDH) activities were visualized from fresh frozen sections of indicated mouse muscle. Scale bars, 100 µm. Biologically independent animals: *n* = 4, 2, 2 (**a**; Sesn1, Sesn2 and Sesn3 analyses, respectively), *n* = 5, 4 (**b**, **d**, **e**; WT and TKO, respectively), *n* = 4, 3 (**c**, PGC1α, SDHA and SDHB; WT and TKO, respectively), *n* = 5, 4 (**c**, all other proteins; WT and TKO, respectively). Error bars, s.e.m. *P < 0.05, **P < 0.01 or ***P < 0.001 from a two-tailed student’s *t* test. Molecular weight markers are indicated in kDa.

Compared to other types of muscle, Sestrin expression of all three forms, especially the muscle-specific isoform Sesn1, was higher in the soleus muscle (SOL), which is enriched with oxidative type I fibers (Fig. 5a). Interestingly, in SOL, type I fiber content was significantly decreased in TKO muscle, while type II fiber content was increased (Fig. 5b). Levels of PGC1α, an essential factor for mitochondrial biogenesis, were also reduced in TKO muscle (Fig. 5c). Markers for mitochondrial complexes, including SDHA, SDHB, NDUFS3, ATP5B, and UQCRFS1, were all significantly decreased in TKO muscle compared to WT (Fig. 5c).

These phenotypes were more pronounced in gastrocnemius (GTN) and quadriceps (QUAD) muscles; expression levels of PGC1α/β, PGC1 target proteins and mitochondrial enzymes were all strongly reduced in TKO tissues (Fig. 5d, e), and the same trend was observed in quantified mRNA levels (Supplementary Fig. 6a) and histological assessment of mitochondrial complex activities (Fig. 5f, g). Muscle mass was also reduced in both SOL and GTN (Supplementary Fig. 6b–d); however, specific force production normalized for the reduced muscle cross-sectional area was comparable between WT and TKO muscle tissues (Supplementary Fig. 6e), suggesting that the exercise deficits in TKO mice are not due to the impairment of muscle contractility. Transmission electron microscopy of GTN muscle showed that the overall microstructure of sarcomeres and mitochondria are not noticeably different between WT and TKO mice (Supplementary Fig. 6f, g). These results suggest that TKO defects in exercise metabolism can be primarily attributed to decreased mitochondrial biogenesis and subsequent reduction in oxidative capacity of muscle tissue.

### Sesn1 expression is induced during myotube differentiation

**Fig. 6:**
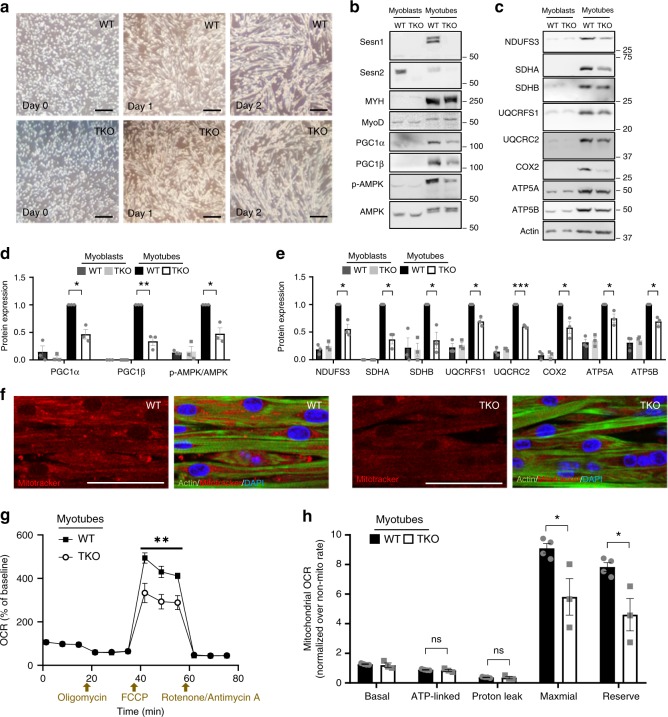
Sestrin is required for maximal oxidative capacity of muscle mitochondria. **a** Myoblasts isolated from WT and TKO mice were differentiated into myotubes. Light microscopy examination of myotube cultures after indicated days of differentiation. Scale bar, 200 µm. **b**–**e** Immunoblotting (**b**, **c**) and densitometry (**d**, **e**; values relative to WT myotube levels) of indicated proteins in WT and TKO myoblasts before (left) and after (right) myotube differentiation. (**f**) Phalloidin (green), mitotracker (red) and DAPI (blue) staining of WT and TKO myotubes. Scale bar, 50 µm. **g** Oxygen consumption rate (OCR) of WT and TKO myotubes, normalized by corresponding baseline OCR, after treatment with oligomycin, carbonyl cyanide-4-phenylhydrazone (FCCP) and rotenone/antimycin A. **h** Indicated mitochondrial OCR was calculated from (**g**), normalized by non-mitochondrial OCR. Biologically independent replicates: *n* = 3 (**d**, **e**), *n* = 4, 3 (**g**, **h**; WT and TKO, respectively). Error bars, s.e.m. *P < 0.05, **P < 0.01, ***P < 0.001 or ns, non-significant from a two-tailed student’s *t* test. Molecular weight markers are indicated in kDa.

We assessed if these metabolic phenotypes of TKO mice could be replicated in isolated myotubes. WT and TKO myoblasts were isolated from hind limb muscles of mice that were not subjected to voluntary wheel running, cultured, and differentiated to form myotubes in vitro. Both WT and TKO myoblasts differentiated well into myotubes, characterized by elongated myofiber morphology, at a similar rate (Fig. 6a). Both WT and TKO myoblasts expressed MyoD (Fig. 6b), but only differentiated myotubes expressed skeletal muscle-specific myosin heavy chain protein MYH (Fig. 6b). Interestingly, although Sesn1 was almost undetectable in myoblasts, it was prominently expressed in differentiated myotubes (Fig. 6b). Sesn2, however, was expressed more in myoblasts and less in myotubes (Fig. 6b). Sesn3 was undetectable in both myotubes and myoblasts, consistent with previous findings that they were expressed at very low levels in muscle (Fig. 5a). This is consistent with former findings that Sesn1 is the predominant Sestrin isoform in muscle tissue^16^.

### TKO myotubes have reduced mitochondrial biogenesis

As observed in intact muscle (Fig. 5), mitochondrial biogenesis was also attenuated in TKO myotubes. During myotube differentiation, WT myoblasts upregulated both PGC1α and PGC1β expression (Fig. 6b), as well as expression of mitochondrial complex proteins including NDUFS3 (Complex I), SDHA/B (Complex II), UQCRFS1, UQCRC2 (Complex III), COX2 (Complex IV), and ATP5A/B (Complex V) (Fig. 6c). This is consistent with former findings that myotube differentiation involves extensive expansion of mitochondrial mass and volume^31,32^, associated with PGC1α/β upregulation and mitochondrial remodeling^33–35^. Interestingly, this process was substantially attenuated in myoblasts isolated from TKO mice; subsequently, TKO myotubes exhibited strongly reduced expression of PGC1α/β proteins, as well as all mitochondrial complex proteins (Fig. 6b–e). Consistent with this, Mitotracker staining of WT and TKO myotubes confirmed that mitochondrial content was generally reduced in TKO myotubes compared to WT (Fig. 6f). These results indicate that differentiated TKO myotubes have decreased mitochondrial biogenesis compared to WT.

### TKO myotubes have reduced AMPK activation

AMPK is one of the most important downstream targets of Sestrins^13,20,21^. Because AMPK regulates PGC1α/β both transcriptionally and post-transcriptionally^36–41^ and because AMPK-mediated PGC1α/β regulation is prominent in myotubes and muscle tissue^36,41^, AMPK may provide a link between Sestrins and PGC1α/β. Consistent with former reports^33,42^, activating phosphorylation of AMPK was increased during myotube differentiation (Fig. 6b, d). However, the differentiation-associated AMPK phosphorylation was attenuated in TKO myotubes (Fig. 6b, d), consistent with the role of Sestrin1 in upregulating AMPK. TKO muscle tissues also exhibited lower AMPK activation compared to WT muscle tissues (Supplementary Fig. 7a–c), although due to substantial animal-to-animal variations, the difference did not reach statistical significance. These results suggest that Sestrins regulate mitochondrial biogenesis through the AMPK-PGC1α/β axis.

### TKO myotubes have reduced reserve respiratory capacity

Through mitochondrial respirometry using Seahorse, we assessed if the reduction of mitochondrial biogenesis in TKO myotubes can precipitate defects in oxidative metabolism (Fig. 6g). Interestingly, basal mitochondrial oxygen consumption rates, as well as specific ATP-linked and proton leak-associated rates, were comparable between WT and TKO myotubes (Fig. 6h). However, the TKO myotubes exhibited strong impairments in maximal respiratory capacity and reserve respiratory capacity (Fig. 6h), indicating that the mitochondrial defects of TKO myotubes are specific to the reserve respiratory capacity. These results suggest that mitochondrial capacity in the TKO muscle was only sufficient for basal metabolism, but not robust enough to afford extensive energy production and oxygen consumption during heavy exercise training. This is consistent with our findings from whole animal respirometry analyses where basal respiration rate was relatively unchanged while exercise-induced maximum respiration rate was severely reduced (Fig. 4). These findings further support the mitochondrial nature of TKO phenotypes in exercise metabolism.

### Sestrin overexpression upregulates mitochondrial biogenesis

We tested whether the benefits of Sestrins can be observed through a gain-of-function study. Consistent with the results of Sestrin in regulating mitochondrial metabolism, Sestrin overexpression in cultured myotubes strongly increased expression of PGC1α/β and its target genes such as mitochondrial enzymes (Supplementary Fig. 8a, b). This was accompanied by corresponding signaling pathway effects such as AMPK activation, TORC1 inhibition and TORC2 upregulation (Supplementary Fig. 8a). These results confirm that Sestrin is sufficient to upregulate mitochondrial biogenesis.

### PGC1 is critical for Sestrin to produce exercise benefits

**Fig. 7:**
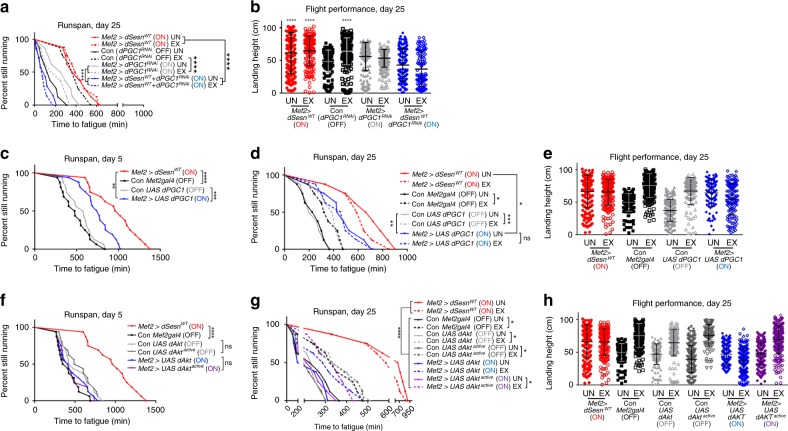
PGC1 is critical for endurance-extending effects of exercise and Sestrin. Flies expressing indicated transgenic components were analyzed by runspan (**a**, **c**, **d**, **f**, **g**) and flight performance (**b**, **e**, **h**) assays at indicated days. See Fig. 2 for details of how individual strains were prepared. *Mef2* *>* *dSesn*^*WT*^ (ON) and Con *Mef2gal4* (OFF) data from the same cohort are shown as a reference. Biologically independent animal groups: *n* = 8, 8, 8, 8, 8, 8, 7, 7 (**a**, upper to lower; groups of 20 flies), *n* = 16 (**c**, **f**; groups o**f** 20 flies), *n* = 8 (**d**, **g**; groups of 20 flies). Biologically independent animals: *n* = 227, 172, 164, 209, 227, 235, 138, 126 (**b**, left to right), *n* = 211, 202, 207, 196, 182, 128, 104, 133 (**e**, left to right), *n* = 211, 202, 207, 196, 138, 168, 223, 115, 146, 221, 168, 205 (**h**, left to right). Error bars, s.d. *P < 0.05, **P < 0.01, ***P < 0.001, ****P < 0.0001 or ns, non-significant from a log-rank test (runspan) or two-tailed student’s *t* test (all other assays).

These results indicate that the PGC1-mitochondrial biogenesis pathway is another important downstream target of the exercise-Sestrin axis, in addition to the TORC1/2 signaling pathway. Also in *Drosophila*, *Drosophila PGC1* (*spargel* or *dPGC1*) expression was reduced in exercised *dSesn*^*−/−*^ mutants (Supplementary Fig. 8c), suggesting that Sestrin regulation of PGC1 is conserved in *Drosophila*. Furthermore, silencing *dPGC1* in fly muscle almost completely inhibited the effect of exercise and Sestrin in extending running endurance (Fig. 7a) and flight performance (Fig. 7b). These results confirm that PGC1, regulated through the Sestrin-AMPK pathway, is an important effector of exercise.

Since both AKT and PGC1 were essential for the effect of exercise and Sestrins in muscle (Figs. 2, 7a, b), we tested if activation of these signaling components is sufficient for mimicking the effect of exercise and Sestrin. Overexpression of dPGC1 was sufficient to extend running endurance in both young (Fig. 7c) and old (Fig. 7d) flies, consistent with our former study^43^. The effect of dPGC1 was stronger than that of exercise; however, weaker than that of Sestrin overexpression (Fig. 7d). dPGC1 overexpression also improved flight performance (Fig. 7e), indicating that PGC1 activation alone could reproduce the effect of exercise although its effect was less pronounced when compared to the effect of Sestrin.

Unlike PGC1, transgenic overexpression of wild-type AKT or constitutively-active AKT, which can respectively produce weak^44^ and strong^45^ activation of the AKT pathway, did not improve running endurance or flight performance (Fig. 7f–h). Therefore, even though AKT signaling was essential for Sestrin-mediated extension of running endurance and flight performance (Fig. 2), activation of the AKT pathway may not be sufficient to mimic the effect of exercise and Sestrin (Fig. 7f–h). However, previous reports indicated that AKT activation in muscle could be sufficient to improve muscle function^46,47^, so it is possible that our transgenic modulation experiments somehow missed the appropriate level of AKT activation to produce beneficial effects and rather produced too much or too little activation.

## Discussion

The current study examined the role of Sestrin in exercise metabolism. In both mice and flies, Sestrin-family proteins were essential for obtaining beneficial effects from exercise. Sestrin-deficient animals did not improve their metabolism or extend endurance even after exercise training. In contrast, Sestrin overexpression mimicked many aspects of exercise training. Specifically, two of Sestrin’s downstream target pathways, the AKT and PGC1 pathways were important for the beneficial effects of Sestrin and exercise.

Sestrin upregulates AKT by activating its upstream kinase TORC2. Sestrin can activate TORC2 through direct association^26,28^ or indirectly through AMPK^21,25^, which was recently shown to phosphorylate and activate TORC2^48^. Sestrin-mediated AMPK activation can lead to upregulation of PGC1 through both transcriptional and post-transcriptional mechanisms^36–41^. The AKT pathway regulates insulin-dependent glucose and fat utilization and maintenance of muscle mass^49,50^, while PGC1 boosts mitochondrial biogenesis and increases oxidative capacity for muscle metabolism^51,52^. Therefore, Sestrin-mediated control of these pathways can mimic the exercise effect without producing some side effects that can result from modulating a single pathway. For instance, while mediating TORC2 activation, which can lead to TORC1 upregulation, Sestrins inhibit TORC1 through AMPK^21^ and GATOR^22–24^. Therefore, some detrimental consequences of TORC1 hyperactivation, such as autophagy inhibition, could be prevented when Sestrin is activated. Likewise, TORC2/AKT activation and TORC1 inhibition can both inactivate PGC1 and reduce mitochondrial biogenesis^53–55^, which would have negative consequences on muscle homeostasis. However, Sestrin can upregulate AMPK signaling^21^, which can subsequently upregulate PGC1 and mitochondrial biogenesis^36–41^. Therefore, Sestrin can produce beneficial effects of TORC1/2 regulation without detrimental effects on PGC1 signaling. Concomitant upregulation of AKT and PGC1 can also cooperate to preserve muscle mass and functionality by inhibiting FoxO, in the context of muscle atrophy and degeneration^56^. Therefore, Sestrin seems to be uniquely poised to coordinate multiple signaling pathways in response to exercise, which can collectively produce many beneficial effects in muscle.

It is also interesting to note that PGC1 inhibition combined with Sestrin overexpression led to worse endurance than PGC1 inhibition alone (Fig. 7a). Similarly, AKT inhibition combined with Sestrin overexpression also led to reduced endurance performance compared to AKT inhibition alone (Fig. 2g). It is possible that, in PGC1- and AKT-inhibited conditions, Sestrin’s other downstream outputs, such as TORC1 inhibition, somehow produced unexpected side effects that interfered with endurance and exercise effects. For instance, since balanced activities of TORC1, PGC1, and AKT pathways are all critical for structural and functional integrity of muscle, it is possible that concomitant inhibition of multiple signaling components, such as TORC1 and PGC1 (Fig. 7a) or TORC1 and AKT (Fig. 2g), produced strong negative impacts on muscle homeostasis and functionality. These observations further support the idea that Sestrin is an important integrator of various signaling pathways, and its effects cannot be fully replicated by modulation of a single downstream output.

Although the role of Sestrin in mediating exercise on healthspan was prominent and robust, the effect of Sestrin modulation on lifespan was not so obvious. Despite strong physiological defects and severely impaired exercise responses, the lifespan of *dSesn*^*−/−*^ flies was only slightly reduced compared to WT flies (Supplementary Fig. 9a). Even though maximum lifespan was reduced, median lifespan was comparable between WT and *dSesn*^*−/−*^ flies. Muscle-specific overexpression of Sestrin, which produced robust extension of endurance and muscle AKT signaling, also did not significantly affect lifespan and rather slightly reduced median lifespan (Supplementary Fig. 9b). It should be noted that, in both humans and mice, endurance exercise training improves healthspan but is not effective for extending maximal lifespan^57,58^. Also in flies, exercise training improved running capacity and flight performance^27^, but did not increase longevity^59^. These results indicate that Sestrin is a specific regulator of healthspan, not longevity.

Considering that Sestrins are induced by a wide variety of stresses^15^, including mechanical and oxidative stresses^60,61^ that are associated with exercise training, it is plausible that exercise induces mild stresses that upregulate Sestrins. Indeed, former studies^17–19^ as well as the current work showed that chronic exercise upregulated Sestrin expression. A number of stress-inducible transcription factors, such as p53, FoxO, ATF4, XBP1, c/EBPβ, HIFα, AP-1, and Nrf2, are involved in Sestrin regulation;^15,62–66^ therefore, it is possible that one or more of these factors are involved in exercise-induced Sestrin upregulation. Interestingly, we found that expression of Sestrin1, the predominant Sestrin isoform in muscle tissue^16^, was almost undetectable in myoblasts but strongly induced during myotube differentiation. Expression of Sestrin2, which shows a more ubiquitous expression pattern across tissues^16^, was slightly decreased during myotube differentiation. Therefore, it is also possible that Sestrin expression is regulated through a stress-independent mechanism in this context.

In summary, our current study highlights the role of Sestrin in mediating the metabolic benefits of exercise. Using mice and *Drosophila*, phylogenetically distant species, we showed that Sestrin’s role in mediating exercise, increasing physical endurance, and improving metabolism is highly conserved across the animal kingdom. Therefore, Sestrin may serve as a promising therapeutic molecule for obtaining exercise-like benefits such as improving mobility and metabolism.

## Methods

### *Drosophila* strains and culture conditions

WT, *dSesn*^*XP4*^ (*UAS-dSesn*) and *dSesn*^*8A11*^ (*dSesn*^*−/−*^) were previously produced in Exelixis *w*^*1118*^ background^14^. *Mef2-Gal4* (#27390), *MHC-GS-Gal4* (#43641), *ap-Gal4* (#3041), *UAS-dAKT* (#8191), *UAS-dAKT*^*RNAi*^ (#33615), *UAS-dRictor*^*RNAi*^ (#36699), *UAS-dSin1*^*RNAi*^ (#32371), *UAS-4E-BP*^*RNAi*^ (#36667), *UAS-dTSC2*^*RNAi*^ (#34737), *UAS-dPGC1*^*RNAi*^ (#33915), and fly lines with an attP landing platform were obtained from the Bloomington *Drosophila* Stock Center (BDSC). *UAS-dSesn*^*RNAi-A*^ (#38481) and *UAS-dSesn*^*RNAi-B*^ (#104365) were obtained from the Vienna Drosophila RNAi Center (VDRC). *UAS-dPGC1* is a kind gift from Dr. David Walker (UCLA), and *UAS-myr-dAKT* (constitutive active AKT) is from Dr. Michelle Bland (University of Virginia). *dSesn* cDNA^14^ of wild-type (*dSesn*^*WT*^) and C86S (*dSesn*^*CS*^)-, D424A (*dSesn*^*DA*^)- or D423A/D424A (*dSesn*^*DDAA*^)-mutated forms were attached to an N-terminal 3 × -FLAG tag, and cloned into a pUAST-attB vector. C86, D423 and D424 in dSesn correspond to C125, D406 and D407 in Sesn2. The constructs were microinjected into embryos of the attP (#24486 from BDSC) strain, which has a PhiC31 integrase insertion on the X chromosome and an attP landing platform on the second chromosome. The transgene insertion was identified by presence of the *mini-white* marker. The flies were cultured on standard cornmeal-agar medium (for strain maintenance and breeding) or 10% sugar-yeast medium (for post-development husbandry, exercise training and lifespan assay) with humidity (70%), temperature (25^o^C) and light (12/12 h light/dark cycle) control.

### Exercise training in *Drosophila*

Cohorts of at least 680 flies were collected under light CO_2_ anesthesia within 2 h of adult eclosion and separated into vials containing 20 flies. Flies were then separated into two groups of more than 340 flies: exercised and unexercised groups. Both unexercised and exercised groups of flies were placed on the exercise training device at the same time to control for exercise-independent environmental factors. Every 15 s, the exercise device drops the vials of flies to induce an innate negative geotaxis response in a repetitive manner. Although exercised flies can run to the top of the vial, unexercised flies were prevented from running by a foam stopper placed low in the vial. Daily exercise time was gradually increased to generate a ramped program that can improve mobility in flies. For all experiments in this study, males were used for all analyses as they are more responsive to exercise training compared to females.

The exercise training was performed at the same time of day each day shortly after lights-on and did not exceed more than 3 h. Sleep is disturbed during the period of training, but the chosen period typically consists of the lowest amount of sleep to minimize the sleep disruption. Any effects seen from the minimal disruption will also be found in the unexercised controls, because they are also placed on the machine at the same time; therefore, sleep deprivation likely does not account for any of the differences seen. More complete description of the exercise protocol, as well as detailed protocols for other analyses such as runspan and flight performance assays, can be found in our recent publication^67^. All biochemical analyses, including western blot and quantitative RT-PCR were performed on flies after more than 24 h of rest after a final exercise bout. Therefore, the observed molecular changes are not from acute effects but from chronic and long-term effects of exercise.

### Running endurance analysis in *Drosophila*

Eight vials of flies from each cohort were subjected to the runspan analysis at two time points: once on day 5 and once on day 25 of adulthood. For each session, the flies were placed on the Power Tower exercise machine^27^ and made to climb until they no longer responded to the negative geotaxis stimulus. Monitored at 15 min intervals, a vial of flies was visually determined to be fatigued when five or fewer flies could climb higher than 1 cm after three consecutive drops. Runspans used a minimum of 8 vials containing 20 flies each. Each vial was plotted as a single datum. Runspan graphs with fewer data points indicate that two or more vials were scored as fatigued at the same time. Each experiment was performed in duplicate or triplicate, and runspans were scored blindly when possible. The time from the start of the assay to the time of fatigue was recorded for each vial, and the data analyzed using log-rank analysis in GraphPad Prism (San Diego, CA, USA).

### Flight performance assays in *Drosophila*

Duplicate or triplicate cohorts of at least 120 flies were aged and/or exercise trained in narrow vials housing groups of 20 age-matched siblings. Acrylic sheeting with paintable adhesive was placed in the flight tube, and fly cohorts were ejected into the apparatus to record flight performance and subsequent landing height after release. Fly cohorts were introduced to the flight tester one vial at a time using a gravity-dependent drop tube in order to reduce variability. After a full cohort of flies was captured on the adhesive, the sheeting was removed to a white surface in order to digitally record the landing height of each fly. Flies with damaged wings were censored from final analysis to control for mechanical stress not related to training performance. Images were analyzed using ImageJ. Landing height was averaged and compared in Prism using a two-tailed student *t* test.

### Controls for genetic and environmental factors in *Drosophila*

For *dSesn* knockout experiments, *dSesn*^*8A11/8A11*^ flies were compared to Exelixis *w*^*1118*^ flies, from which the mutant allele was generated^14^. For *dSesn* RNAi experiments, adult progeny was collected from the same vial over a 72-h time period, and randomly split into control (OFF) and experimental (ON) groups. ON group received 100 μM mifepristone (Cayman Chemical, Ann Arbor, MI), which activates gene switch (GS) driver, while OFF group received same trace amount of vehicle solution (70% ethanol) until the experimental endpoint. For *dSesn* overexpression experiments, the attP (#24486 from BDSC) strain, which was used for generating *UAS-dSesn*^*WT*^ and mutant *dSesn* strains, were instead crossed to *Mef2-Gal4* and used as a genetic background control. These flies were used for both exercise and lifespan assays.

Exercise-independent environmental factors were controlled for by exposing unexercised flies to the same environment in all respects except that unexercised flies were prevented from running as described above.

### Mouse strains and rearing conditions

All mice used in this study are in the C57BL/6 background. *Sesn1*^−/−^ mice^68^ and *Sesn2*^*−/−*^/*Sesn3*^*−/−*^ mice^25^, both in the C57BL/6 background, were interbred to produce TKO mice. As formerly reported^69^, the TKO mice showed reduced viability and semi-sterility, but considerable number of mice survived up to adulthood without any gross developmental abnormalities. Mice were maintained in filter-topped cages and were given free access to autoclaved regular chow diet (LFD; 5L0D, Lab Diet) or HFD (S3282, Bio-Serv, when indicated) and water at the University of Michigan according to National Institutes of Health (NIH) and institutional guidelines. We complied with all relevant ethical regulations for animal testing and research. All experiments were approved by the University of Michigan Institutional Animal Care & Use Committee (Protocol numbers: PRO00007710, PRO00006772, PRO00006689, PRO00006206, and PRO00005712).

### Voluntary wheel running

Voluntary running experiments were performed at the University of Michigan Frankel Cardiovascular Center – Physiology Phenotyping Core, according to their established protocols. In brief, running wheels (Comfort Wheel; Central Garden and Pet) were introduced to allow mice to voluntarily exercise. 6-month-old WT (n = 4) and *Sesn1*^*−/−*^ (n = 5) male mice (Fig. 3), 1-year old WT (n = 3; *Sesn1*^*+/−*^ littermate control) and *Sesn1*^*−/−*^ (n = 4) male mice (Supplementary Fig. 2), and 5-month-old WT (n = 5) and *Sesn1*^*−/−*^/*Sesn2*^*−/−*^/*Sesn3*^*−/−*^ (TKO, n = 4) mice (Fig. 4) were subjected to voluntary wheel running. Running wheel activity was monitored daily through cyclocomputer (Cateye) for 3 weeks. After the period, mice were kept on the wheels for 4–6 additional weeks until additional metabolic analyses including glucose tolerance tests, respirometry and insulin response studies were completed.

### Glucose tolerance test and insulin response studies

For glucose tolerance tests, mice were starved for 6 h, and blood was drawn from a tail nick at the indicated time points after i.p. injection of glucose (1 g kg^−1^ body weight), and blood glucose was instantly measured with a one-touch ultra glucose meter (Lifescan, Inc.). For an acute insulin response study, mice were kept under surgical-plane anesthesia using isoflurane, and skeletal muscle was harvested from each leg before and after injection of insulin (0.8 U kg^−1^ body weight). The tissues were snap frozen and used for subsequent analyses such as immunoblotting.

### Metabolic cage

Metabolic cage, body composition, and exercise respirometry experiments were performed at the Michigan Mouse Metabolic Phenotyping Center Core, according to their established protocols. In brief, oxygen consumption (VO_2_), carbon dioxide production (VCO_2_), spontaneous movements, and food intake were measured using the Comprehensive Laboratory Monitoring System (CLAMS, Columbus Instruments). After measuring body weight, each mouse was placed into the sealed chambers (7.9″ × 4″ × 5″) individually. The study was carried out continuously for 96 h, in an environmental room set at 20–23 °C with 12–12 h (6:00PM–6:00AM) dark-light cycles. During this time, food and water were provided to the animals through the feeding and drinking devices equipped inside the chamber. The amount of food consumed by each animal was monitored through a precision balance installed under the chamber. A standard gas (20.5% O_2_ and 0.5% CO_2_ in N_2_) was used to calibrate the system before each experiment. VO_2_ and VCO_2_ samplings were done sequentially for 5 s in a 10 min interval. Spontaneous activity was recorded every second in *X* and *Z* dimensions. The air flow rate was adjusted to keep the oxygen differential around 0.3% at resting conditions. RER was calculated as VCO_2_∙VO_2_^−1^.

### Body composition

Body weight, fat mass, and lean body mass were measured using an NMR analyzer (Minispec LF90II, Bruker Optics), maintained according to the manufacturer’s recommendation. Conscious mice were put into the measuring tube with minimal restraint, and the individual measurements took less than 2 min.

### Exercise respirometry

VO_2_ and VCO_2_ were measured using the CLAMS instrument described above. Before the study, the mice were each placed into the treadmill chambers to acclimate them to the treadmill environment. For two days prior to the study, the mice were individually put into the same treadmill for 30 min each day. Mice were weighed prior to the running test. They were then individually placed into the sealed treadmill chambers (305 × 51 × 44 mm^3^). The slope of the treadmill was set at 25° to the horizontal. The measurements were only carried out between 9:00AM and 3:00PM on each day. During this time, the animals were run on the treadmills one at a time and the treadmill was wiped clean between each test. As described above, the CLAMS system was routinely calibrated before the experiment using the standard gas. VO_2_ and VCO_2_ in each chamber were sampled continuously every 5 s. The air flow rate through the chambers was set at 0.50 LPM. RER was calculated as VCO_2_∙VO_2_^−1^. Total glucose oxidation and fatty acid oxidation are calculated, respectively, based on the values of VO_2_ and VCO_2_ using equations 1.69∙VO_2_ - 1.69∙VCO_2_ and 4.57∙VCO_2_ - 3.23∙VO_2_, respectively. The mice were all ran under the same standard treadmill schedule, which was: 30 min baseline recording, 5 min at 5 m min^−1^, 9 m min^−1^, 12 m min^−1^ and 15 m min^−1^, and then 2 min at 17–47 m min^−1^ increasing by 2 m min^−1^. Mice were run until they are exhausted. Exhaustion was qualified by a mouse sitting on the shocker (1.60 mA, 120 V, 3 Hz) for five consecutive seconds, at which point the shocker was shut off and treadmill schedule stopped. Then, 15–20 min of recovery data were recorded.

### Primary myoblast culture and differentiation

Primary myoblasts were isolated from hind limb muscles of 2 month-old WT and TKO male mice. The isolated myoblasts were cultured in myoblast growth media (F-10 media, 20% FBS, 10 ng ml^−1^ basic fibroblast growth factor, Penicillin-Streptomycin). For differentiation into myotubes, when primary myoblasts were around 95% confluency, differentiation media (DMEM, 2% horse serum) was treated for 5 days. Phase contrast microscope images were taken under an inverted microscope attached to a digital camera during the course of differentiation. For fluorescent imaging, fully differentiated myotubes were incubated with 100 nM Mitotracker CMX-ROS (M7512, Invitrogen) for 30 min at 37 °C. After fixation with 4% paraformaldehyde and permeabilization with 0.2% Triton X-100, the cells were treated with 6.6 µM Alexa Fluor 488-conjugated phalloidin (A12379, Invitrogen) for 45 min and 1 μg mL^−1^ DAPI for 10 s at room temperature then imaged under Leica SP5 confocal microscope.

### Measurements of mitochondrial oxygen consumption

Mitochondrial oxygen consumption rate was measured through the XFe96 Extracellular Flux Analyzer (Seahorse Biosciences) according to the manufacturer’s recommendation. Oxygen consumption rate (OCR, moles min^−1^) was measured as an index of mitochondrial function. Initially, baseline rates were measured, and then, 1 μM oligomycin, 0.25 μM FCCP and 0.5 μM rotenone/antimycin A (XF cell mito stress test kit from Agilent Technologies, 103015-100) were injected sequentially through the ports of the Seahorse flux assay kit cartridge. The rates were measured at three consecutive time points. A line diagram of the OCR measurements was shown after normalization with baseline rates. Mitochondria-specific respiration rates were also calculated. Basal mitochondrial respiration was derived by subtracting the rotenone rate from the baseline rate, ATP-linked respiration by subtracting the oligomycin rate from the baseline rate, proton leak respiration by subtracting the rotenone rate from the oligomycin rate, maximal mitochondrial respiration by subtracting the rotenone rate from the FCCP rate, and reserve mitochondrial respiration by subtracting the baseline rate from the FCCP rate. All these mitochondrial respiration values were normalized by non-mitochondrial respiration, which is same as the rotenone rate.

### C2C12 myoblast culture and differentiation

C2C12 cells were originated from ATCC (CRL-1772) and cultured in DMEM supplemented with 10% FBS and Penicillin-Streptomycin. Cells were tested negative for Mycoplasma contamination in PCR-based analysis using these primers: F: GTGGGGAGCAAA(C/T)AGGATTAGA, and R: GGCATGATGATTTGACGTC(A/G)T. For differentiation, when cells were at 80–90% confluence, growth media was replaced with differentiation media (DMEM, 2% horse serum) for 5 days. Differentiated C2C12 cells were verified through their myotube morphology, and subjected to adenoviral infection.

### Adenoviral procedures

Flag-tagged full-length human Sesn2 was cloned into pACCMV-I shuttle vector, and then assembled into the adenoviral backbone at the University of Michigan Vector Core (Ad-SESN2). GFP-expressing adenoviruses (Ad-GFP), constructed in the same way, were used as a negative control. For dose-dependent infection experiments for C2C12 myotubes, total amount of viral particles used for infection was kept constant, and the ratio between Ad-SESN2 and Ad-GFP was proportionally changed according to the infection scheme. At 36 h after infection, harvested cells were subsequently utilized for immunoblotting or quantitative RT-PCR.

### Antibodies

dSesn, Sesn1, and Sesn2 antibodies were generated from rabbits and guinea pigs using GST-fusion proteins^14,25^. All in-house antibodies were affinity purified using PVDF-immobilized target proteins and confirmed through knockout tissue lysates. Commercial Sesn3 antibodies (Abcam, ab97792) were also used to detect Sestrins. Tubulin (T5168) antibodies are from Sigma. Actin (JLA20), Wingless (4D4), type I MHC (BA-D5), type IIA MHC (SC-71) and type IIB MHC (BF-F3) antibodies are from Developmental Studies Hybridoma Bank (DSHB). Phospho-Thr398 *Drosophila* S6k (9209), phospho-Thr389 S6k (9234), phosphor-Thr172 AMPKα (phospho-Thr184 *Drosophila* AMPK; 2535), AMPKα (2532), phospho-Ser79 ACC (3661), ACC (3676), Flag (2368), phospho-Ser505 *Drosophila* Akt1 (4054), phospho-Ser473 mouse Akt1 (9271) and mouse/*Drosophila* Akt antibodies (4691) are from Cell Signaling Technology. PGC1α (sc-517380), PGC1β (sc-373771), MYH (skeletal muscle myosin heavy chain; sc-32732), MyoD (sc-377460), NDUFS3 (sc-374282), SDHA (sc-390381), SDHB (sc-271548), COX2 (sc-514489), UQCRFS1 (sc-271609), UQCRC2 (sc-390378), ATP5A (sc-136178), ATP5B (sc-55597) and S6K (sc-8418) antibodies are from Santa Cruz Biotechnology. For immunoblotting, antibodies were diluted at 1:200 for Santa Cruz antibodies and at 1:1,000 for all other antibodies. For immunostaining, antibodies were diluted at 1:500 (type IIA MHC antibody) or 1:100 (all other antibodies).

### Muscle histology

Whole mouse soleus and gastrocnemius muscles were snap frozen in O.C.T compound (Tissue-Tek) using isopentane-cooled in liquid nitrogen, and cut into 10-µm-thick cryosections. To determine muscle fiber type, muscle cryosections were permeabilized in 0.5% Triton X-100 in PBS, treated in MOM blocking solution (Vector laboratories, BMK-2202), according to the manufacturer’s instructions. The sections were incubated overnight at 4 °C with primary monoclonal antibodies against type I myosin heavy chain (MHC) (BA-D5, mouse IgG2b), type IIA MHC (SC-71, mouse IgG1) and type IIB MHC (BF-F3, mouse IgM). The primary monoclonal antibodies were detected with goat anti-mouse secondary antibodies against IgG2b (Alexa 350, A-21140), IgG1 (Alexa 555, A-21127), and IgM (Alexa 647, A-21238), obtained from Invitrogen. To visualize extracellular matrix, wheat germ agglutin (WGA) lectin conjugated to AlexaFluor 488 (Life Technologies, W11261) was used. Type IIX muscle fibers were detected by the absence of immunofluorescent signal. Fluorescence images were obtained through Nikon A1 confocal microscope. The sections of gastrocnemius were stained for NADH-TR (complex I) and succinate dehydrogenase (SDH, complex II) through the following method. For histochemical detection of mitochondrial complex I activity, sections were incubated for 30 min at 37 °C in freshly prepared 1 mg ml^−1^ nitroblue tetrazolium and 1 mg ml^−1^ NADH in 100 mM Tris (pH 7.6) solution. For detecting mitochondrial complex II activity, the sections were dried at the room temperature for 10 min and were rehydrated with PBS (pH 7.2), and then incubated in a complex II assay solution containing 50 mM phosphate buffer (pH 7.4), 50 mM succinic acid, and 0.5 mg mL^−1^ nitroblue tetrazolium (NBT) at 37 °C in a humidity chamber for 30 min. The sections were washed in distilled water, dried, and then mounted in glycergel mounting medium (Dako). All histological sections were imaged under a light microscope (Meiji).

### Transmission electron microscopy

For transmission electron microscopy, samples were fixed in 2.5% glutaraldehyde in 0.1 M Sorensen’s buffer, pH 7.4, overnight at 4 °C. The next day, samples were rinsed twice in Sorensen’s, fixed in 1% osmium tetroxide in Sorensen’s for 1 h, and rinsed in double distilled water. The samples were then dehydrated in ascending concentrations of ethanol, 10 min each, rinsed twice in acetone, and embedded in epoxy resin. Resin blocks were cut to 70 nm ultra-thin sections and stained with uranyl acetate and lead citrate. Sections were imaged on a JEOL 1400 + electron microscope at 80 keV with Hamamatsu ORCA-HR digital camera system.

### Contractile force

Contractile properties of skeletal muscle were examined through the following method^70^. Intraperitoneal injection of tribromoethanol (400 mg kg^−1^) was used to anesthetize the mice, and anesthesia was maintained by supplemental injections of tribromoethanol throughout the procedure. Contractile properties of gastrocnemius (GTN) muscle were measured in situ. The whole GTN muscle was isolated from surrounding muscle and connective tissue of anesthetized mice, and the distal tendon was severed and secured to the lever arm of a servomotor (model 305B, Aurora Scientific). Muscles were activated via stimulation of the tibial nerve by platinum wire electrodes. Stimulation voltage was adjusted to produce maximum force, typically between 5 and 10 V. With muscles held at optimum length for force production, trains of 0.2 ms stimulus pulses were applied. Pulse frequency was increased until a maximum force was reached. Contractile properties of soleus (SOL) muscles were measured in vitro. Each SOL muscle was removed from the animal and placed in a horizontal bath containing buffered mammalian Ringer solution (137 mM NaCl, 24 mM NaHCO_3_, 11 mM glucose, 5 mM KCl, 2 mM CaCl_2_, 1 mM MgSO_4_, 1 mM NaH_2_PO_4_, and 0.025 mM turbocurarine chloride) maintained at 25 °C. The solution was bubbled with 95% O_2_/5% CO_2_ to maintain pH 7.4. One tendon was tied to a force transducer (model BG-50, Kulite Semiconductor Products Inc.) and the other tendon was tied to a fixed post. Muscles were stimulated between two platinum plate electrodes, and a maximum force was measured as described above for GTN muscles. After all force measurements, muscle mass was measured and total fiber cross-sectional area (CSA) was calculated by dividing the muscle mass by the product of fiber length (determined from previously established ratios of optimum muscle length to fiber length) and muscle density, 1.06 g per cm^2^. Maximum specific force (SOL, kN per m^2^; GTN, N per cm^2^) was calculated for each muscle by dividing maximum force by CSA.

### Immunoblotting

For immunoblotting, tissue lysates were boiled in 1X SDS sample buffer for 5 min, separated by SDS-PAGE, transferred to PVDF membranes and probed with primary antibodies and then with horseradish peroxidase-conjugated secondary antibodies. Chemiluminescence was detected using LAS4000 (GE) systems. Uncropped immunoblot images were provided as a Source Data file.

### Immunostaining of imaginal discs

Third instar wandering-stage larvae of the indicated genotypes were collected, rinsed and dissected in phosphate-buffered saline (PBS) for immunostaining^14^. Imaginal disc complexes were fixed in Brower Fix (0.15 M PIPES pH 6.9, 3 mM MgSO_4_, 1.5 mM EGTA, 1.5% NP-40) mixed with one-third volume of 8% methanol-free formaldehyde for 3 h at 4 °C. After washing in PBT (PBS with 0.1% Tween-20), the tissues were incubated in 1X Western blocking reagent (Roche) diluted in PBT for 1 h at room temperature. The tissues were then incubated overnight at 4 °C with primary antibodies in 1X Western blocking reagent. The tissues were then washed with PBT, and incubated for 3 h at room temperature with fluorophore-conjugated secondary antibodies (Invitrogen) in 1X Western blocking reagent. After washing with PBT, the tissues were rinsed with PBS and mounted in ProLong Gold anti-fade reagent (Invitrogen). Samples were examined under a Nikon A1 confocal microscope.

### Quantitative RT-PCR

Total RNA was extracted using the Trizol system (Invitrogen). RNA was treated with DNase I (Thermo Fisher, 18068015) and reverse transcribed using MMLV reverse transcriptase (Thermo Fisher, 28025013) with random hexamers (Thermo Fisher, N8080127). Relative transcript amounts were measured by the StepOnePlus Real Time PCR system (Applied Biosystems), using iQ SYBR Green Supermix (Bio-rad, 1708884). All mRNA expression data were normalized to the *Actb* level (mouse) or the *rp49* level (*Drosophila*). Following primer pairs were used.

*Myod1* F, CCACTCCGGGACATAGACTTG

*Myod1* R, AAAAGCGCAGGTCTGGTGAG

*Myog* F, GAGACATCCCCCTATTTCTACCA

*Myog* R, GCTCAGTCCGCTCATAGCC

*Myf5* F, AAGGCTCCTGTATCCCCTCAC

*Myf5* R, TGACCTTCTTCAGGCGTCTAC

*Myh7* (*Mhc-I*) F, ACTGTCAACACTAAGAGGGTCA

*Myh7* (*Mhc-I*) R, TTGGATGATTTGATCTTCCAGGG

*Myh2* (*Mhc-IIa*) F, AAGTGACTGTGAAAACAGAAGCA

*Myh2* (*Mhc-IIa*) R, GCAGCCATTTGTAAGGGTTGAC

*Myh1* (*Mhc-IIx*) F, GCGAATCGAGGCTCAGAACAA

*Myh1* (*Mhc-IIx*) R, GTAGTTCCGCCTTCGGTCTTG

*Myh4* (*Mhc-IIb*) F, TTGAAAAGACGAAGCAGCGAC

*Myh4* (*Mhc-IIb*) R, AGAGAGCGGGACTCCTTCTG

*Pgc1a* F, TATGGAGTGACATAGAGTGTGCT

*Pgc1a* R, CCACTTCAATCCACCCAGAAAG

*Pgc1b* F, TCCTGTAAAAGCCCGGAGTAT

*Pgc1b* R, GCTCTGGTAGGGGCAGTGA

*Ppara* F, AGAGCCCCATCTGTCCTCTC

*Ppara* R, ACTGGTAGTCTGCAAAACCAAA

*Esrra* F, CTCAGCTCTCTACCCAAACGC

*Esrra* R, CCGCTTGGTGATCTCACACTC

*Tfam* F, ATTCCGAAGTGTTTTTCCAGCA

*Tfam* R, TCTGAAAGTTTTGCATCTGGGT

*Cytc* F, CAGCTTCCATTGCGGACAC

*Cytc* R, GGCACTCACGGCAGAATGAA

*Ckmt* F, ACACCCAGTGGCTATACCCTG

*Ckmt* R, CCGTAGGATGCTTCATCACCC

*Cox5a* F, GCCGCTGTCTGTTCCATTC

*Cox5a* R, GCATCAATGTCTGGCTTGTTGAA

*Mtco2* F, AATTGCTCTCCCCTCTCTACG

*Mtco2* R, GGTTTTAGGTCGTTTGTTGGGAT

*Cpt1b* F, GCACACCAGGCAGTAGCTTT

*Cpt1b* R, CAGGAGTTGATTCCAGACAGGTA

*Oxct1* F, CATAAGGGGTGTGTCTGCTACT

*Oxct1* R, GCAAGGTTGCACCATTAGGAAT

*Mdh1* F, TTCTGGACGGTGTCCTGATG

*Mdh1* R, TTTCACATTGGCTTTCAGTAGGT

*Idh3a* F, TGGGTGTCCAAGGTCTCTC

*Idh3a* R, CTCCCACTGAATAGGTGCTTTG

*Actb* F, CAAAAGCCACCCCCACTCCTAAGA

*Actb* R, GCCCTGGCTGCCTCAACACCTC

*dPGC1* F, GGATTCACGAATGCTAAATGTGTTCC

*dPGC1* R, GATGGGTAGGATGCCGCTCAG

*rp49* F, ACGTTGTGCACCAGGAACTT

*rp49* R, CCAGTCGGATCGATATGCTAA

### Reporting summary

Further information on research design is available in the Nature Research Reporting Summary linked to this article.

## Supplementary information

Supplementary Information

Reporting Summary

## Data Availability

The datasets generated and analyzed during the current study, as well as materials unique to this study, are available from the corresponding authors on reasonable request. The source data underlying Figs. 1b–j, 2a–l, 3a–f, 4a–l, 5a–e, 6b–e, g, h, 7a–h and Supplementary Figs. 1a–f, i, j, 2a–d, 3–9 are provided as a Source Data file.

## Source data

Source Data

## References

[1] Cartwright T (2007). ‘Getting on with life’: the experiences of older people using complementary health care. Soc. Sci. Med..

[2] Finlayson ML, Peterson EW (2010). Falls, aging, and disability. Phys. Med. Rehabil. Clin. N. Am..

[3] Nagatomo I, Kita K, Takigawa M, Nomaguchi M, Sameshima K (1997). A study of the quality of life in elderly people using psychological testing. Int J. Geriatr. Psychiatry.

[4] Bo H, Zhang Y, Ji LL (2010). Redefining the role of mitochondria in exercise: a dynamic remodeling. Ann. N. Y Acad. Sci..

[5] Gibala M (2009). Molecular responses to high-intensity interval exercise. Appl. Physiol. Nutr. Metab..

[6] Ventura-Clapier R, Mettauer B, Bigard X (2007). Beneficial effects of endurance training on cardiac and skeletal muscle energy metabolism in heart failure. Cardiovasc. Res..

[7] Cutler RG, Mattson MP (2006). The adversities of aging. Ageing Res. Rev..

[8] Ascensao A, Lumini-Oliveira J, Oliveira PJ, Magalhaes J (2011). Mitochondria as a target for exercise-induced cardioprotection. Curr. Drug Targets.

[9] Kennedy RL, Chokkalingham K, Srinivasan R (2004). Obesity in the elderly: who should we be treating, and why, and how?. Curr. Opin. Clin. Nutr. Metab. Care.

[10] Corcoran MP, Lamon-Fava S, Fielding RA (2007). Skeletal muscle lipid deposition and insulin resistance: effect of dietary fatty acids and exercise. Am. J. Clin. Nutr..

[11] Erickson KI, Kramer AF (2009). Aerobic exercise effects on cognitive and neural plasticity in older adults. Br. J. Sports Med..

[12] Egan B, Zierath JR (2013). Exercise metabolism and the molecular regulation of skeletal muscle adaptation. Cell Metab..

[13] Lee JH, Budanov AV, Karin M (2013). Sestrins orchestrate cellular metabolism to attenuate aging. Cell Metab..

[14] Lee JH (2010). Sestrin as a feedback inhibitor of TOR that prevents age-related pathologies. Science.

[15] Budanov AV, Lee JH, Karin M (2010). Stressin’ Sestrins take an aging fight. EMBO Mol. Med..

[16] Peeters H (2003). PA26 is a candidate gene for heterotaxia in humans: identification of a novel PA26-related gene family in human and mouse. Hum. Genet.

[17] Zeng Nina, D'Souza Randall F., Figueiredo Vandre C., Markworth James F., Roberts Llion A., Peake Jonathan M., Mitchell Cameron J., Cameron-Smith David (2017). Acute resistance exercise induces Sestrin2 phosphorylation and p62 dephosphorylation in human skeletal muscle. Physiological Reports.

[18] Liu X, Niu Y, Yuan H, Huang J, Fu L (2015). AMPK binds to Sestrins and mediates the effect of exercise to increase insulin-sensitivity through autophagy. Metabolism.

[19] Lenhare L (2017). Physical exercise increases Sestrin 2 protein levels and induces autophagy in the skeletal muscle of old mice. Exp. Gerontol..

[20] Ho A, Cho CS, Namkoong S, Cho US, Lee JH (2016). Biochemical basis of Sestrin physiological activities. Trends Biochem. Sci..

[21] Budanov AV, Karin M (2008). p53 target genes sestrin1 and sestrin2 connect genotoxic stress and mTOR signaling. Cell.

[22] Chantranupong L (2014). The Sestrins interact with GATOR2 to negatively regulate the amino-acid-sensing pathway upstream of mTORC1. Cell Rep..

[23] Parmigiani A (2014). Sestrins inhibit mTORC1 kinase activation through the GATOR complex. Cell Rep..

[24] Kim, J. S. et al. Sestrin2 inhibits mTORC1 through modulation of GATOR complexes. *Sci. Rep.*10.1038/srep09502 (2015). 10.1038/srep09502 PMC4377584 25819761

[25] Lee JH (2012). Maintenance of metabolic homeostasis by Sestrin2 and Sestrin3. Cell Metab..

[26] Tao R, Xiong X, Liangpunsakul S, Dong XC (2015). Sestrin 3 protein enhances hepatic insulin sensitivity by direct activation of the mTORC2-Akt signaling. Diabetes.

[27] Sujkowski A, Wessells R (2018). Using *Drosophila* to understand biochemical and behavioral responses to exercise. Exerc. Sport Sci. Rev..

[28] Byun JK (2017). A positive feedback loop between Sestrin2 and mTORC2 is required for the survival of glutamine-depleted lung cancer cells. Cell Rep..

[29] Dowling RJ (2010). mTORC1-mediated cell proliferation, but not cell growth, controlled by the 4E-BPs. Science.

[30] Edvardsen Elisabeth, Hem Erlend, Anderssen Sigmund A. (2014). End Criteria for Reaching Maximal Oxygen Uptake Must Be Strict and Adjusted to Sex and Age: A Cross-Sectional Study. PLoS ONE.

[31] Kislinger T (2005). Proteome dynamics during C2C12 myoblast differentiation. Mol. Cell Proteom..

[32] Miyake Tetsuaki, McDermott John C., Gramolini Anthony O. (2011). A Method for the Direct Identification of Differentiating Muscle Cells by a Fluorescent Mitochondrial Dye. PLoS ONE.

[33] Fortini P (2016). The fine tuning of metabolism, autophagy and differentiation during in vitro myogenesis. Cell Death Dis..

[34] Sin J (2016). Mitophagy is required for mitochondrial biogenesis and myogenic differentiation of C2C12 myoblasts. Autophagy.

[35] Lin Y (2014). PGC-1α is associated with C2C12 myoblast differentiation. Cent. Eur. J. Biol..

[36] Jager S, Handschin C, St-Pierre J, Spiegelman BM (2007). AMP-activated protein kinase (AMPK) action in skeletal muscle via direct phosphorylation of PGC-1alpha. Proc. Natl Acad. Sci. USA.

[37] Lin J, Handschin C, Spiegelman BM (2005). Metabolic control through the PGC-1 family of transcription coactivators. Cell Metab..

[38] Terada S (2002). Effects of low-intensity prolonged exercise on PGC-1 mRNA expression in rat epitrochlearis muscle. Biochem. Biophys. Res. Commun..

[39] Suwa M, Nakano H, Kumagai S (2003). Effects of chronic AICAR treatment on fiber composition, enzyme activity, UCP3, and PGC-1 in rat muscles. J. Appl Physiol. (1985).

[40] Canto C (2009). AMPK regulates energy expenditure by modulating NAD+ metabolism and SIRT1 activity. Nature.

[41] Narkar VA (2008). AMPK and PPARdelta agonists are exercise mimetics. Cell.

[42] Niesler CU, Myburgh KH, Moore F (2007). The changing AMPK expression profile in differentiating mouse skeletal muscle myoblast cells helps confer increasing resistance to apoptosis. Exp. Physiol..

[43] Tinkerhess Martin J., Healy Lindsey, Morgan Matthew, Sujkowski Alyson, Matthys Erin, Zheng Li, Wessells Robert J. (2012). The Drosophila PGC-1α Homolog spargel Modulates the Physiological Effects of Endurance Exercise. PLoS ONE.

[44] Rintelen F, Stocker H, Thomas G, Hafen E (2001). PDK1 regulates growth through Akt and S6K in Drosophila. Proc. Natl Acad. Sci. USA.

[45] Verdu J, Buratovich MA, Wilder EL, Birnbaum MJ (1999). Cell-autonomous regulation of cell and organ growth in *Drosophila* by Akt/PKB. Nat. Cell Biol..

[46] Kim MH (2011). Myogenic Akt signaling attenuates muscular degeneration, promotes myofiber regeneration and improves muscle function in dystrophin-deficient mdx mice. Hum. Mol. Genet..

[47] Marshall JL (2012). Sarcospan-dependent Akt activation is required for utrophin expression and muscle regeneration. J. Cell Biol..

[48] Kazyken Dubek, Magnuson Brian, Bodur Cagri, Acosta-Jaquez Hugo A., Zhang Deqiang, Tong Xin, Barnes Tammy M., Steinl Gabrielle K., Patterson Nicole E., Altheim Christopher H., Sharma Naveen, Inoki Ken, Cartee Gregory D., Bridges Dave, Yin Lei, Riddle Steven M., Fingar Diane C. (2019). AMPK directly activates mTORC2 to promote cell survival during acute energetic stress. Science Signaling.

[49] Manning BD, Cantley LC (2007). AKT/PKB signaling: navigating downstream. Cell.

[50] Schiaffino S, Mammucari C (2011). Regulation of skeletal muscle growth by the IGF1-Akt/PKB pathway: insights from genetic models. Skelet. Muscle.

[51] Handschin C (2007). Skeletal muscle fiber-type switching, exercise intolerance, and myopathy in PGC-1alpha muscle-specific knock-out animals. J. Biol. Chem..

[52] Zechner C (2010). Total skeletal muscle PGC-1 deficiency uncouples mitochondrial derangements from fiber type determination and insulin sensitivity. Cell Metab..

[53] Li X, Monks B, Ge Q, Birnbaum MJ (2007). Akt/PKB regulates hepatic metabolism by directly inhibiting PGC-1alpha transcription coactivator. Nature.

[54] Cunningham JT (2007). mTOR controls mitochondrial oxidative function through a YY1-PGC-1alpha transcriptional complex. Nature.

[55] Bentzinger CF (2008). Skeletal muscle-specific ablation of raptor, but not of rictor, causes metabolic changes and results in muscle dystrophy. Cell Metab..

[56] Sandri M (2006). PGC-1alpha protects skeletal muscle from atrophy by suppressing FoxO3 action and atrophy-specific gene transcription. Proc. Natl Acad. Sci. USA.

[57] Garcia-Valles R (2013). Life-long spontaneous exercise does not prolong lifespan but improves health span in mice. Longev. Healthspan.

[58] Kujala UM (2018). Is physical activity a cause of longevity? It is not as straightforward as some would believe. A critical analysis. Br. J. Sports Med.

[59] Sujkowski A, Bazzell B, Carpenter K, Arking R, Wessells RJ (2015). Endurance exercise and selective breeding for longevity extend *Drosophila* healthspan by overlapping mechanisms. Aging (Albany NY).

[60] Budanov AV (2002). Identification of a novel stress-responsive gene Hi95 involved in regulation of cell viability. Oncogene.

[61] Frank D (2008). Gene expression pattern in biomechanically stretched cardiomyocytes: evidence for a stretch-specific gene program. Hypertension.

[62] Shi X (2016). Sestrin2 induced by hypoxia inducible factor1 alpha protects the blood-brain barrier via inhibiting VEGF after severe hypoxic-ischemic injury in neonatal rats. Neurobiol. Dis..

[63] Ye J (2015). GCN2 sustains mTORC1 suppression upon amino acid deprivation by inducing Sestrin2. Genes Dev..

[64] Saveljeva S (2016). Endoplasmic reticulum stress-mediated induction of SESTRIN 2 potentiates cell survival. Oncotarget.

[65] Kim MG (2015). Regulation of Toll-like receptor-mediated Sestrin2 induction by AP-1, Nrf2, and the ubiquitin-proteasome system in macrophages. Toxicol. Sci..

[66] Shin BY, Jin SH, Cho IJ, Ki SH (2012). Nrf2-ARE pathway regulates induction of Sestrin-2 expression. Free Radic. Biol. Med.

[67] Damschroder, D., Cobb, T., Sujkowski, A. & Wessells, R. *Drosophila* endurance training and assessment of its effects on systemic adaptations. *Bio-protocol* 10.21769/BioProtoc.3037 (2018). 10.21769/BioProtoc.3037 PMC8342081 34532514

[68] Lanna A (2017). A sestrin-dependent Erk-Jnk-p38 MAPK activation complex inhibits immunity during aging. Nat. Immunol..

[69] Peng M, Yin N, Li MO (2014). Sestrins function as guanine nucleotide dissociation inhibitors for Rag GTPases to control mTORC1 signaling. Cell.

[70] Lustgarten MS (2009). Conditional knockout of Mn-SOD targeted to type IIB skeletal muscle fibers increases oxidative stress and is sufficient to alter aerobic exercise capacity. Am. J. Physiol. Cell Physiol..

