# Supplementary material for: Sestrins are evolutionarily conserved mediators of exercise benefits
Source: Nat Commun. 2020 Jan 13;11:190. doi: 10.1038/s41467-019-13442-5 (PMC6955242; doi:10.1038/s41467-019-13442-5)
Supplement: Supplementary file 1 — Supplementary Information [file 41467_2019_13442_MOESM1_ESM.pdf]

Supplementary Information

Sestrins are evolutionarily conserved mediators of exercise benefits

Kim et al.

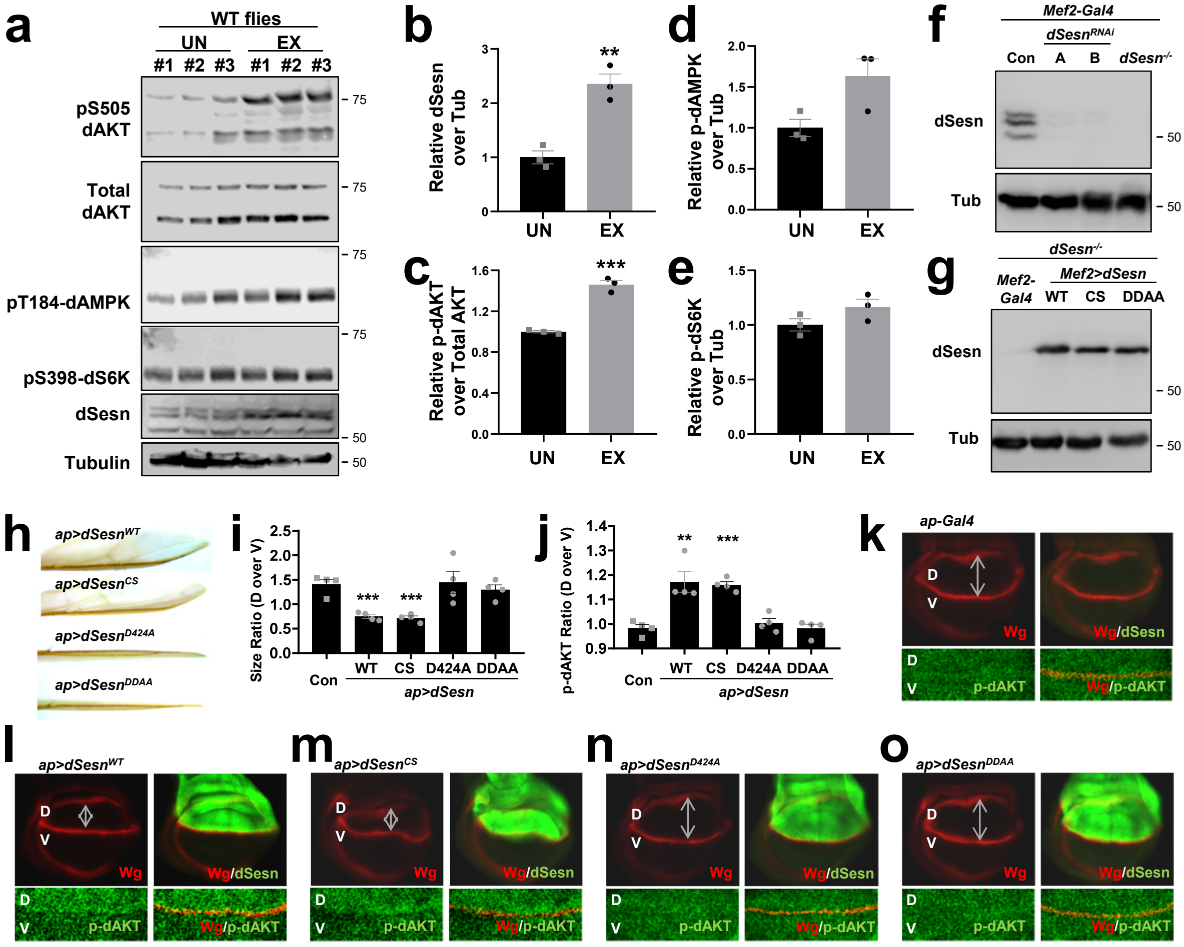

Supplementary Figure 1 | Exercise induces dSesn expression and dAKT activation. (**a**) After a control (UN) or exercise (EX) regimen as described in Fig. 1a, legs and thoraxes of WT flies (n=10 biologically independent flies per each lane) were subjected to immunoblot analyses of indicated signaling markers. (**b-e**) Band intensities were quantified by densitometry (n=3 biologically independent groups, each with 10 biologically independent flies). (**f**, **g**) Legs and thoraxes of WT flies (n=10 biologically independent flies per each lane) expressing muscle-specific dSesn RNAi or *dSesn*-null flies expressing WT and mutant dSesn were analyzed by immunoblotting. S505 of dAKT corresponds to S473 in AKT. T184 of dAMPK corresponds to T172 of AMPK. S398 of dS6K corresponds to S389 in S6K. (**h-o**) dSesn^{WT} and dSesn^{CS}, but not dSesn^{D424A} or dSesn$^{D424/425A(DDAA)}$, suppressed mTORC1-dependent wing growth and activated mTORC2-dependent AKT phosphorylation. (**h**) Anterior views of wing blades with *ap-Gal4*-driven expression of indicated genetic elements. Dorsal sides point upward. Suppression of dorsal tissue growth induces the wing blade bent upward. (**i-o**) Third instar larval wing imaginal discs were stained with Wg, dSesn and phospho-S505-dAKT (p-dAKT) antibodies as indicated. Sestrin transgenes were expressed only in the dorsal compartment through an *apterous* (ap)-*Gal4* driver and UAS enhancer. Dorsal (D) and ventral (V) compartments were labeled. Tissue growth (double-headed arrows, upper panels) and p-dAKT intensities (lower panels) of dorsal compartments were quantified, normalized by ventral values (D over V), and presented as bar graphs (**i, j**; n=4 biologically independent flies). Error bars, s.e.m. ***P<0.001 and **P<0.01 from a two-tailed student's t-test (compared to control). Molecular weight markers are indicated in kDa.

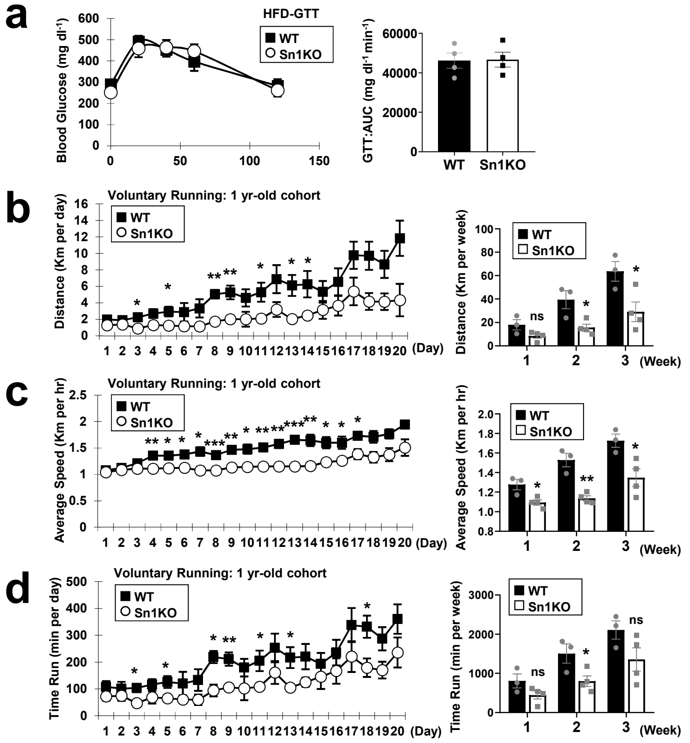

Supplementary Figure 2 | *Sesn1*-knockout mice phenocopy *dSesn*-null flies in exercise response. (**a**) 8-week-old WT (n=4 biologically independent animals) and *Sesn1*$^{-/-}$ (n=4 biologically independent animals; Sn1KO) mice were kept on high-fat diet (HFD) for 12 additional weeks and subjected to glucose tolerance tests (GTT) and area-under-curve (AUC) analyses. (**b-d**) 1-year-old WT (n=3 biologically independent animals; *Sesn1*$^{+/-}$ littermate control) and *Sesn1*$^{-/-}$ (n=4 biologically independent animals) male mice were put in a wheel running cage and assessed for their daily running profile, including daily running distance (Km per day; **b**), average running speed (Km per hour; **c**) and total running time (min per day; **d**). Daily data are shown as line graphs while the weekly pooled data are shown as bar graphs. Error bars, s.e.m. *P<0.05, **P<0.01, ***P<0.001 or ns, non-significant from a two-tailed student's t-test.

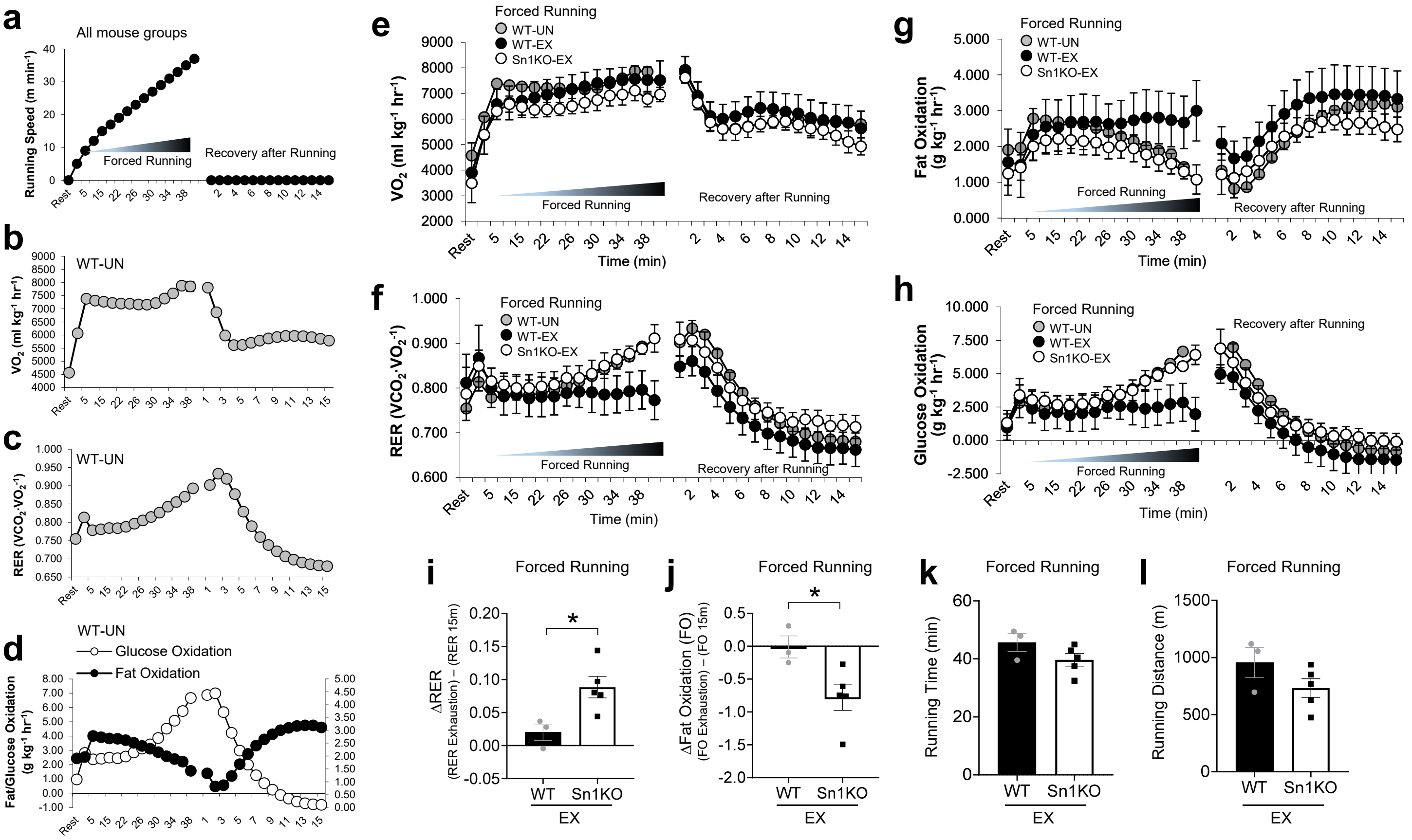

Supplementary Figure 3 | The respiration-improving effect from daily running was attenuated in *Sesn1*$^{-/-}$ mice. (**a-d**) Experimental scheme for exercise calorimetry. Oxygen consumption (VO_2) and carbon dioxide production (VCO_2) were measured from untrained C57BL/6 WT male mice during forced treadmill running (n=56 biologically independent animals, 2-3 months old). The intensity of treadmill running (running speed) was gradually increased until exhaustion, then 15 minutes of recovery data were obtained after the treadmill stopped, according to the schedule depicted in (**a**). VO_2 (**b**) and VCO_2 measurements were used to calculate respiratory exchange ratio (RER, **c**) and glucose and fat oxidation (**d**), as described in the Methods. (**e-l**) 6-month-old WT (n=3 biologically independent animals) and *Sesn1*$^{-/-}$ (n=5 biologically independent animals; Sn1KO) male mice were put in a wheel running cage (EX) for two months, then analyzed by exercise calorimetry. Data from untrained WT mice (WT-UN) were used as a reference. VO_2 (**e**) and VCO_2 measurements were used to calculate respiratory exchange ratio (RER; **f**) and fat (**g**) and glucose (**h**) oxidation. RER differences (ΔRER in **i**) and fat oxidation (FO) differences (ΔFat Oxidation in **j**) was calculated between values from low (the measurement at 15 m min^{-1}) and high (the last measurement of each mouse before exhaustion) exercise intensities of forced treadmill running. Running time (**k**) and distance (**l**) were recorded during forced running. Error bars, s.e.m. *P<0.05 from a two-tailed student's t-test.

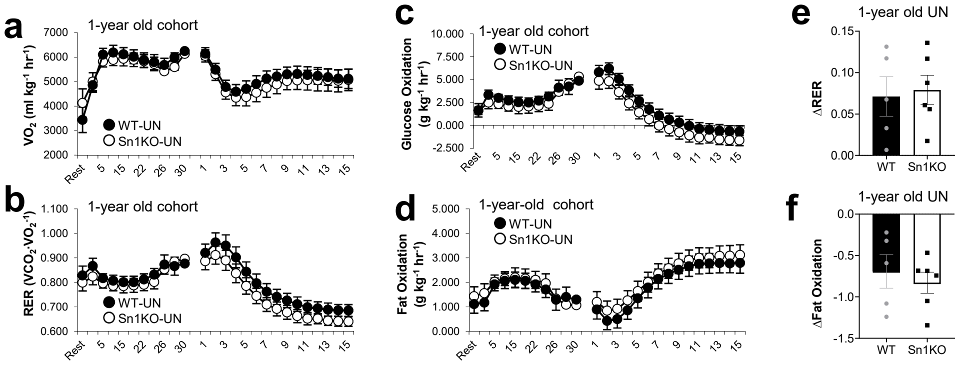

Supplementary Figure 4 | Sesn1 mutation does not alter basal exercise calorimetry. 1-year-old WT (n=5 biologically independent animals) and *Sesn1*$^{-/-}$ (n=6 biologically independent animals) male mice, which were rendered sedentary (UN), were analyzed by exercise calorimetry. VO_2 (**a**) and VCO_2 measurements were used to calculate respiratory exchange ratio (RER; **b**) and glucose (**c**) and fat (**d**) oxidation. RER differences (ΔRER in **e**) and fat oxidation differences (ΔFat Oxidation in **f**) was calculated between values from low (the measurement at 15 m min^{-1}) and high (the last measurement of each mouse before exhaustion) exercise intensities of forced treadmill running. Error bars, s.e.m.

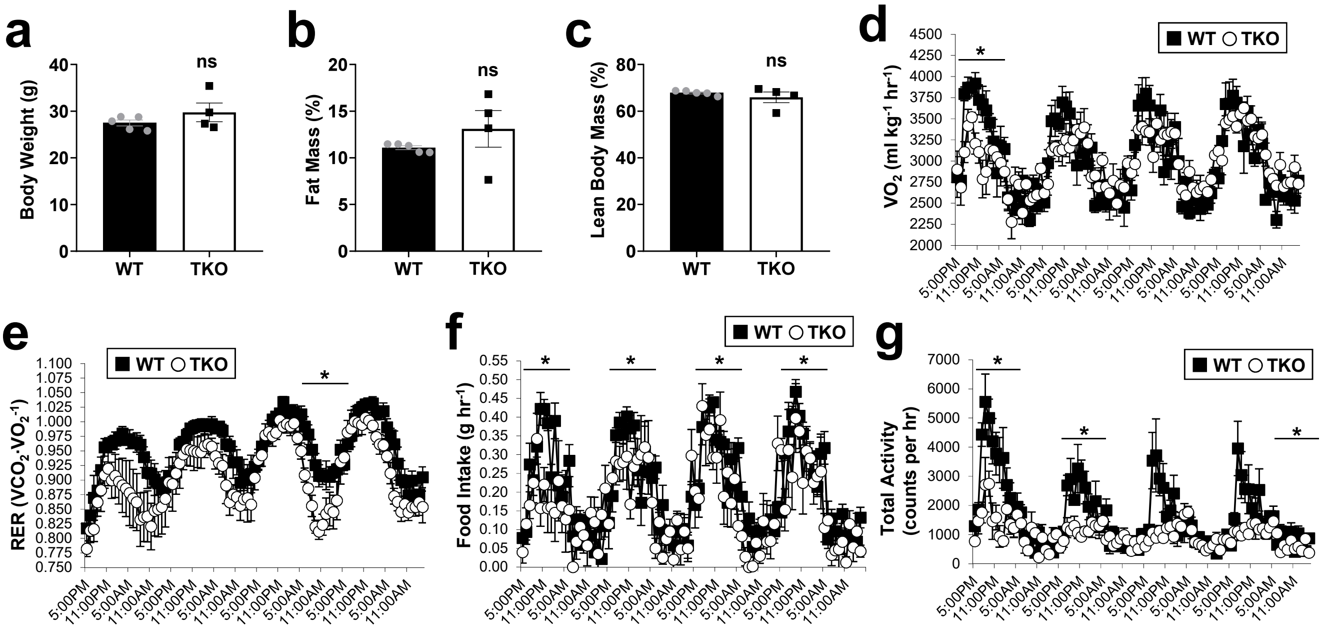

Supplementary Figure 5 | Metabolic profiles of Sesn1-3 triple knockout mice. 5-month-old WT (n=5 biologically independent animals) and *Sesn1$^{-/-}$/Sesn2$^{-/-}$/Sesn3$^{-/-}$* (TKO) mice (n=4 biologically independent animals) were subjected to voluntary wheel running for 1 month. After wheel running, body weight (**a**) and composition (**b, c**) were examined, and oxygen consumption rate (**d**), respiratory exchange ratio (**e**), food intake (**f**) and physical activity (**g**) were monitored in metabolic cages for 4 consecutive days. Error bars, s.e.m. *P<0.05 or ns, non-significant from a two-tailed student's t-test.

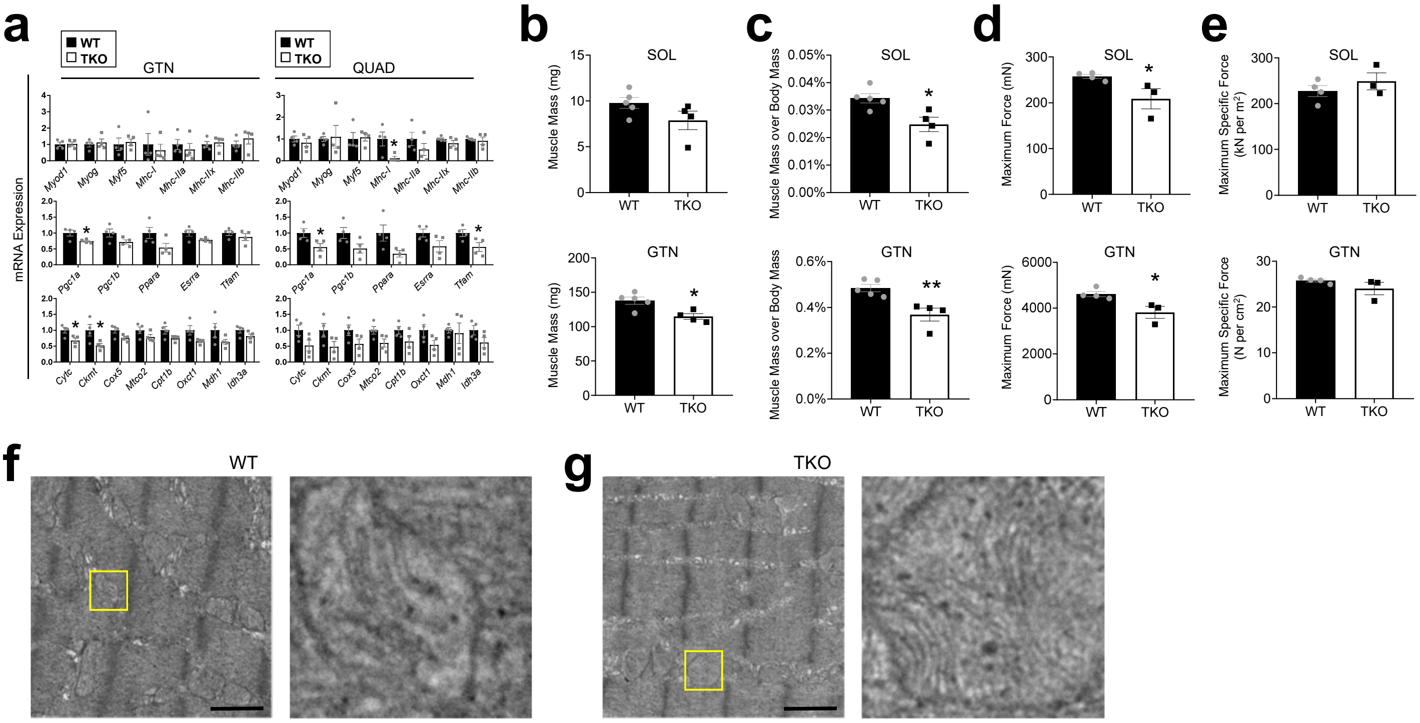

Supplementary Figure 6 | Sestrin controls mitochondrial biogenesis in mouse muscle. (**a**) GTN (gastrocnemius) and QUAD (quadriceps) muscles of WT and *Sesn1*$^{-/-}$/*Sesn2*$^{-/-}$/*Sesn3*$^{-/-}$ (TKO) mice described in Fig. 4 were analyzed by quantitative RT-PCR (n=4 biologically independent animals). (**b-e**) Indicated biophysical properties of muscle were measured from soleus (SOL) and gastrocnemius (GTN) tissues of WT (n=5 biologically independent animals) and TKO (n=4 biologically independent animals) mice. (**f**, **g**) GTN muscles of indicated 2-year-old mice were analyzed through transmission electron microscopy. Boxed areas are magnified in right panels. Scale bars, 1 µm. Error bars, s.e.m. *P<0.05 or **P<0.01 from a two-tailed student's t-test.

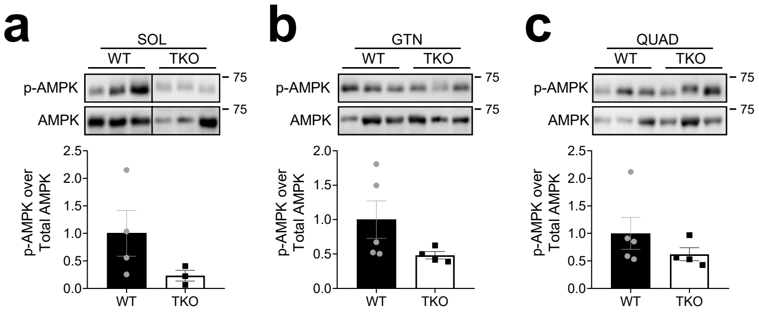

Supplementary Figure 7 | Effects of Sestrin on AMPK activation in mouse muscle tissue. (**a-c**) Activating AMPK phosphorylation (p-AMPK) was examined in soleus (SOL), gastrocnemius (GTN) and quadriceps (QUAD) tissues of WT and TKO mice through immunoblotting (top) and densitometry (bottom). Biologically independent animals: n=4, 3 (**a**; WT and TKO mice, respectively), n=5, 4 (**b**, **c**; WT and TKO mice, respectively). Error bars, s.e.m.

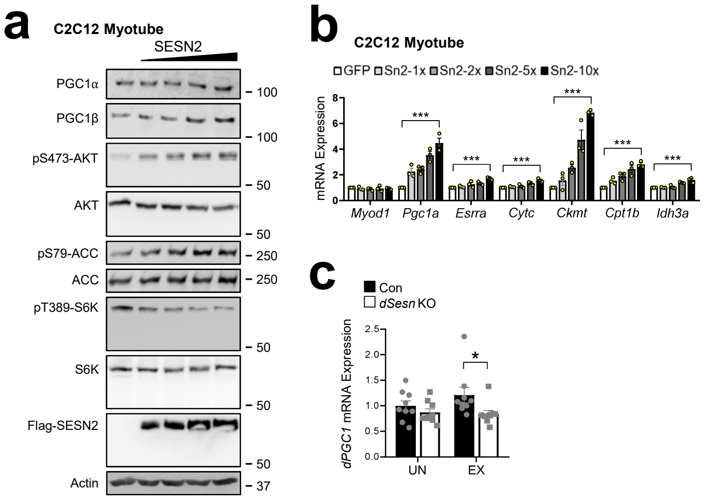

Supplementary Figure 8 | Effects of Sestrin on PGC1 expression. (**a**, **b**) C2C12 myoblasts were differentiated into myotubes and then infected with different doses of Ad-GFP or Ad-SESN2 (Sn2). 36 hours after infection, myotube lysates were subjected to immunoblotting (**a**) and quantitative RT-PCR analyses (**b**, n=3 biologically independent replicates). (**c**) WT and *dSesn*$^{-/-}$ flies were subjected to control (UN) or exercise (EX) treatment and *dPGC1* expression in indirect flight muscle tissue was analyzed through quantitative RT-PCR (n=9 biologically independent groups, each with more than 10 biologically independent flies). *$P<0.05$ or ***$P<0.001$ from a two-tailed student's t-test.

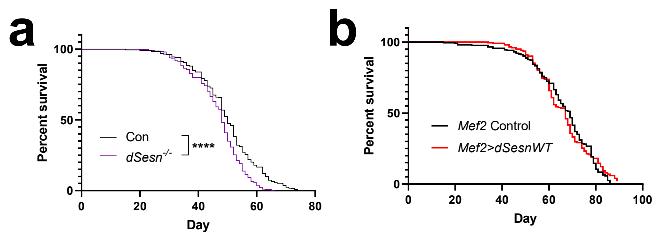

Supplementary Figure 9 | Effects of Sestrin on lifespan of *Drosophila*. (**a**, **b**) Survival for indicated fly strains was monitored from 2 weeks after adult eclosion. Biologically independent flies: n=283, 289, 203, 203 (**a**-Con, **a**-*dSesn*$^{-/-}$, **b**-*Mef2* Control, and **b**-*Mef2>dSesnWT*, respectively). ****P<0.0001 from a log-rank test.
