# Supplementary material for: Sestrins are evolutionarily conserved mediators of exercise benefits
Source: Nat Commun. 2020 Jan 13;11:190. doi: 10.1038/s41467-019-13442-5 (PMC6955242; doi:10.1038/s41467-019-13442-5)
Supplement: Supplementary file 2 — Reporting Summary [file 41467_2019_13442_MOESM2_ESM.pdf]

nature research

Corresponding author(s): Jun Hee Lee
Robert J. Wessells

Last updated by author(s): Oct 12, 2019

Reporting Summary

Nature Research wishes to improve the reproducibility of the work that we publish. This form provides structure for consistency and transparency in reporting. For further information on Nature Research policies, see Authors & Referees and the Editorial Policy Checklist.

Statistics

For all statistical analyses, confirm that the following items are present in the figure legend, table legend, main text, or Methods section.

n/a	Confirmed	
☐	☒	The exact sample size (*n*) for each experimental group/condition, given as a discrete number and unit of measurement
☐	☒	A statement on whether measurements were taken from distinct samples or whether the same sample was measured repeatedly
☐	☒	The statistical test(s) used AND whether they are one- or two-sided<br>*Only common tests should be described solely by name; describe more complex techniques in the Methods section.*
☐	☒	A description of all covariates tested
☒	☐	A description of any assumptions or corrections, such as tests of normality and adjustment for multiple comparisons
☐	☒	A full description of the statistical parameters including central tendency (e.g. means) or other basic estimates (e.g. regression coefficient) AND variation (e.g. standard deviation) or associated estimates of uncertainty (e.g. confidence intervals)
☐	☒	For null hypothesis testing, the test statistic (e.g. *F*, *t*, *r*) with confidence intervals, effect sizes, degrees of freedom and *P* value noted<br>*Give P values as exact values whenever suitable.*
☒	☐	For Bayesian analysis, information on the choice of priors and Markov chain Monte Carlo settings
☒	☐	For hierarchical and complex designs, identification of the appropriate level for tests and full reporting of outcomes
☒	☐	Estimates of effect sizes (e.g. Cohen's *d*, Pearson's *r*), indicating how they were calculated

Our web collection on statistics for biologists contains articles on many of the points above.

Software and code

Policy information about availability of computer code

Data collection	N/A
Data analysis	N/A

For manuscripts utilizing custom algorithms or software that are central to the research but not yet described in published literature, software must be made available to editors/reviewers. We strongly encourage code deposition in a community repository (e.g. GitHub). See the Nature Research guidelines for submitting code & software for further information.

Data

Policy information about availability of data

All manuscripts must include a data availability statement. This statement should provide the following information, where applicable:

- Accession codes, unique identifiers, or web links for publicly available datasets
- A list of figures that have associated raw data
- A description of any restrictions on data availability

The datasets generated and analyzed during the current study, as well as materials unique to this study, are available from the corresponding authors on reasonable request. The source data underlying Figs 1b-j, 2a-l, 3a-f, 4a-l, 5a-e, 6b-e, g, h, 7a-h and Supplementary Figs 1a-f, i, j, 2a-d, 3-9 are provided as a Source Data file.

Field-specific reporting

Please select the one below that is the best fit for your research. If you are not sure, read the appropriate sections before making your selection.

☒ Life sciences ☐ Behavioural & social sciences ☐ Ecological, evolutionary & environmental sciences

Life sciences study design

All studies must disclose on these points even when the disclosure is negative.

Sample size	Sample sizes were designed to be comparable to former exercise studies in flies and mice (e.g. see our former papers PMID: 19517023, 22348115, 29141215, 19776389, 21913219, 25640809).
Data exclusions	Data from accidentally dead animals were excluded from the study.
Replication	All attempts at replication were successful.
Randomization	Control (UN) and exercised (EX) groups were randomly assigned from the same cohort of progenies.
Blinding	All the physiological measurements were recorded in a blinded manner, and later assigned to each experimental group.

Reporting for specific materials, systems and methods

We require information from authors about some types of materials, experimental systems and methods used in many studies. Here, indicate whether each material, system or method listed is relevant to your study. If you are not sure if a list item applies to your research, read the appropriate section before selecting a response.

Materials & experimental systems

n/a	Involved in the study	
☐	☒	Antibodies
☐	☒	Eukaryotic cell lines
☒	☐	Palaeontology
☐	☒	Animals and other organisms
☒	☐	Human research participants
☒	☐	Clinical data

Methods

n/a	Involved in the study	
☒	☐	ChIP-seq
☒	☐	Flow cytometry
☒	☐	MRI-based neuroimaging

Antibodies

Antibodies used	All antibodies used are listed in the methods section, with specific catalog numbers.
Validation	Commercial antibodies were validated by the manufacturer and are commonly used in the field. Antibodies specific to this study were validated through knockout cells and tissues.

Eukaryotic cell lines

Policy information about cell lines

Cell line source(s)	C2C12 cells were originated from ATCC (CRL-1772).
Authentication	Differentiation into myotubes confirmed the authenticity.
Mycoplasma contamination	Negative by PCR
Commonly misidentified lines (See ICLAC register)	*Name any commonly misidentified cell lines used in the study and provide a rationale for their use.*

Animals and other organisms

Policy information about studies involving animals; ARRIVE guidelines recommended for reporting animal research

Laboratory animals	Mice and flies: Strains, genders and ages are specified in the methods and legends.
Wild animals	N/A
Field-collected samples	N/A
Ethics oversight	We complied with all relevant ethical regulations for animal testing and research. All experiments were approved by the University of Michigan Institutional Animal Care & Use Committee (Protocol PRO00007710).

Note that full information on the approval of the study protocol must also be provided in the manuscript.
